# Aggregates and Excitons: Excited-State Behavior of Platinum–Acetylide Two-Photon Chromophore-Doped Ormosil Glasses

**DOI:** 10.1021/acs.jpca.5c05728

**Published:** 2025-10-29

**Authors:** Thomas M. Cooper, Jonathan E. Slagle, Douglas M. Krein, Joy E. Haley

**Affiliations:** † 97042 Materials and Manufacturing Directorate Air Force Research Laboratory, Wright-Patterson Air Force Base, Ohio 45433, United States; ‡ General Dynamics Information Technology, Dayton, Ohio 45420, United States

## Abstract

We probe the excited-state dynamics of a platinum–acetylide chromophore dissolved in ormosil glasses in the concentration range of 0.1–400 mM to gain a better understanding of how the environment of the dye reflects upon the overall kinetics observed. At 0.1 mM, ground-state absorption, fluorescence, excited-state absorption (ESA), and triplet ESA reproduce solution behavior. Above ≥10 mM, a weak 485 nm ground-state band appears, consistent with a nominally forbidden S_0_ → T_1_ transition, and steady-state emission shows quenched fluorescence with enhanced phosphorescence. Following 355 nm flash photolysis, high-concentration samples initially exhibit triplet ESA identical to the 0.1 mM case, but a blue-shifted triplet ESA develops at longer delays; direct excitation of the 485 nm band yields the same blue-shifted spectrum, confirming aggregation effects. Kinetically, the 0.1 mM sample displays a single triplet lifetime, whereas ≥10 mM samples require two. The shorter lifetime at all loadings follows a Freundlich adsorption dependence, consistent with monomer binding to ormosil sites, while the longer lifetime is attributed to aggregation. Ultrafast transient absorption (TA) resolves two ESA bands whose energy separation and relative areas suggest intramolecular exciton coupling between ligand-localized transitions. Fitting the data with exciton theory gives the interligand transition-dipole angle and the excitonic splitting; both evolve with concentration and pump–probe delay, reflecting symmetry breaking, intersystem crossing, and charge-transfer reorganization. At ∼1 mM, the time-dependent band separation is consistent with excimer formation, whereas no excimer signatures are observed at ≥10 mM. These results establish a quantitative structure–dynamics–concentration relationship: aggregation and ormosil-induced microphase separation create coexisting free and aggregated populations that modulate exciton coupling (dipole geometry and splitting) and govern the triplet photophysics.

## Graphical abstract

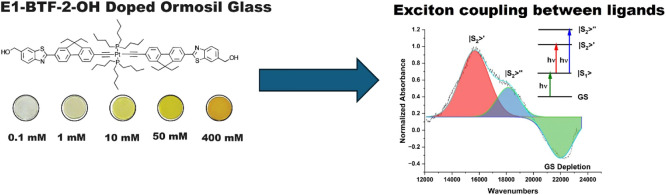

## Introduction

Solid-state materials containing nonlinear dyes have many photonic applications.^1,2^ Their real and imaginary refractive index components are a function of input light intensity leading to nonlinear refraction and absorption.^3^ The nonlinear index relates to the third-order nonlinear optical susceptibility, concentration, and molecular parameters.^3^ A well-behaved dye dissolved in a solid matrix should mimic dilute solution behavior over a wide concentration range and have a long triplet state lifetime, good photostability, and minimum intermolecular excited-state interactions, including excimers, quenching, and aggregation.

In this work, we investigate the photophysics of the platinum acetylide E1-BTF-2OH doped in ormosil glasses with a concentration range of 0.1–400 mM, as shown in Figure 1. Ormosil glasses are excellent solid-state host materials for nonlinear optical dyes.^4^ They have been functionalized with a variety of dopants, including laser,^5^ photochromic,^6^ nonlinear,^7,8^ and holographic^9^ dyes. They have high broadband optical quality and good mechanical properties and are an alternative to organic polymers as host materials. We have previously studied the behavior of E1-BTF-2-OH dissolved in PMMA^10^ and epoxy^11^ matrix.

**Figure 1:**
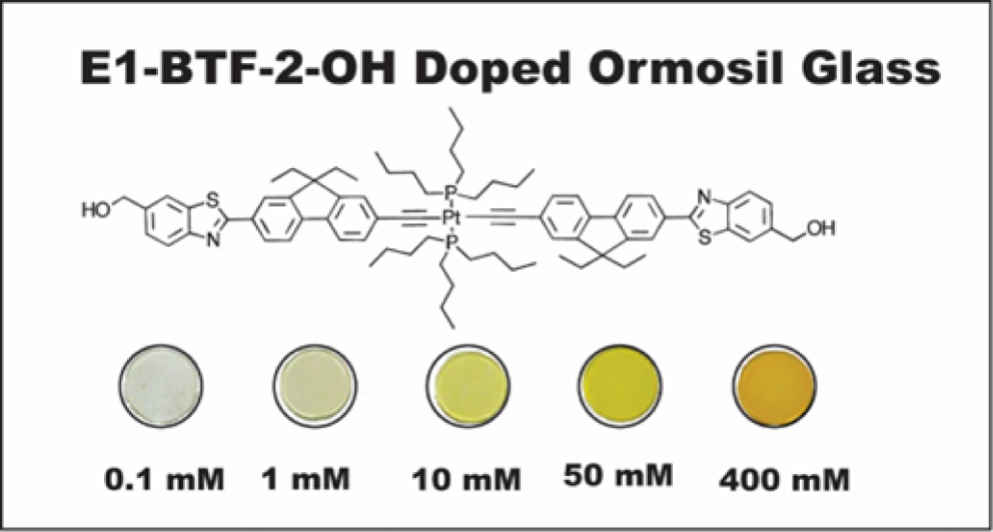
Photograph of samples prepared in this study ranging from 0.1 to 400 mM in concentration. The chemical structure of E1-BTF-2-OH is also shown.

The E1-BTF-2OH chromophore has two ligands attached to the central platinum atom and is a quadrupolar A−π–D−π–A type chromophore whose S_1_ state has MLCT character. Previous structure–property investigations of platinum acetylides include two-photon spectroscopy,^12,13^ triplet state,^14^ ligand length,^15,16^ electron-donating and -withdrawing effects,^14^ neat chromophore liquids,^17^ and liquid crystals.^18^ E1-BTF-2-OH has a large two-photon absorption cross section and strong spin–orbit coupling affecting linear and nonlinear optical behavior.^19^

The optical spectra of chromophore aggregates give rich information about intermolecular excited-state interactions between monomer units.^20–26^ The various types of aggregates (J, H, and herringbone) have spectroscopic band shapes and transition dipoles influenced by the aggregate structure and host environment. Aggregation-induced phosphorescence enhancement (AIPE) refers to changes in the nonradiative decay rate and emission quantum yield resulting from chromophore aggregation and solid-state host environment.^27,28^ Similarly, intramolecular interactions between ligands have been investigated.^29–31^ Excited-state symmetry breaking (ESSB) refers to nominally symmetric conformationally floppy chromophores whose spectroscopic behavior reflects a conformation distribution sensitive to flexible vs rigid excited-state potential energy surfaces, host environment effects, and molecular aggregation.^32–45^

We measured linear ground-state absorption, steady-state emission, time-resolved triplet-state spectra, and ultrafast-transient-absorption (TA) spectra, which exhibited aggregation effects in the high-concentration samples. We show concentration effects in the ground-state absorption spectra, where a new absorption band appears at high concentration. Similarly, AIPE is observed in the emission spectra, and the triplet-state absorption spectra of the high-concentration samples show a new band. The TA spectra of E1-BTF-2-OH exhibit two exciton bands arising from intramolecular interactions between two ligand transition dipoles. The TA spectra are a function of concentration and time, resulting from molecular conformation change, ormosil host reorganization, intersystem crossing, chromophore aggregation, and charge transfer processes. The insights from this spectroscopic behavior will enable the development of solid-state optical materials where there are minimum aggregation effects at high concentration.

## Materials and Methods

All chemicals used for synthesis were of reagent grade and used without further purification unless otherwise noted. All reactions were carried out under an argon atmosphere with freshly distilled solvents. E1-BTF-2-OH shown in Figure 1 was synthesized according to previous methods analogous to the parent compound E1-BTF with a slight variation in initial ligand used.^19^ We prepared a series of ormosil glasses, also sometimes referred to as sol gel, from methyltriethoxysilane incorporating the dye E1-BTF-2-OH.^46,47^ Briefly, to a round-bottom flask with a magnetic stir bar, were added triethoxymethyl silane (TEMS), distilled water, and 3.5 M HCl. This solution was warmed to 40 °C for approximately 4 h or until the cloudy solution became clear. Once clear, the solution was warmed to 78 °C and left overnight. This solution was then evaporated down to afford a slightly milky syrup that was then diluted with THF and any residual visible water removed via a pipet, dried with MgSO_4_, filtered, and then evaporated down to give a syrup that had a dried weight percentage of 55–60% (1.00 g of syrup was placed in an aluminum dish and then put in the drying oven for 1 h, which resulted in a dried weight of 0.55–0.60 g). To generate a boule from the syrup, it was placed in a PTFE casting dish with a lid. To this was added the desired chromophore dissolved in THF after passing it through a 1 μm PTFE syringe filter. This mixture was thoroughly mixed in a vortex and then degassed in a vacuum chamber. Once degassed, 4-aminopropyltriethoxysilane (APTES) was introduced via pipet and thoroughly mixed with a spatula. The container was loosely sealed and placed in a 40 °C oven until all THF vapor was removed, which was approximately 2 days. To fully cure the boule, the now hardened boule was removed from the PTFE container and placed in an 80 °C oven for an additional 2 days. Once fully cured, the boule was sectioned on a diamond wire saw, and each section was polished to 1 mm thickness with an optical finish on a lapping wheel. The samples have dye concentrations ranging from 0.1, 1, 10, 50, and 400 mM. The process for verifying the absence of phase separation or crystallization was done by observing under a microscope as well as looking at the ground-state absorption spectra and comparing them to data obtained for the chromophore in solution. If there was significant crystallization or phase separation, we would observe scattering in the absorption measurements, and this was not seen.

Ground-state absorption spectra, emission spectra, emission lifetime via time-correlated single photon counting (TCSPC), picosecond-time-scale transient absorption spectra (TA), and microsecond-time-scale transient absorption spectra (MTA) were measured as previously published.^18^ In all measurements, to ensure the linearity of the experiment, precautions were taken. For ground-state absorption measurements, a pinhole was used in both the reference and sample beams to ensure linearity. For emission measurements, both steady-state and time-resolved, samples were placed in a front-face geometry to minimize issues with the excitation beam passing through the sample. There is likely some reabsorption of the emitted light by the sample, but this technique tries to minimize these effects. Lastly, in the MTA and TA measurements, no changes were made, but only data was obtained at the wavelengths that the probe beam was able to transmit through the sample. Delta absorbance level values were kept below 0.4 by controlling laser power of the excitation beam to ensure linearity.

## Results and Discussion

### Linear Spectroscopy

Figure 2A shows ground-state absorption spectra for the 0.1 mM sol–gel sample, E1-BTF-2-OH ligand, and the complex dissolved in benzene. The absorption spectrum of the 0.1 mM sol–gel sample shows no evidence of free ligand and resembles the spectrum of the E1-BTF-2-OH complex in benzene. The spectrum of the 0.1 mM sample has a slight blue shift in the peak maximum compared to the spectrum of the dye dissolved in benzene. The blue shift can be attributed to the interactions between the dye and its environment, including cavity formation, hydrogen bonding, electrostatic, and dispersion forces.^48^

**Figure 2:**
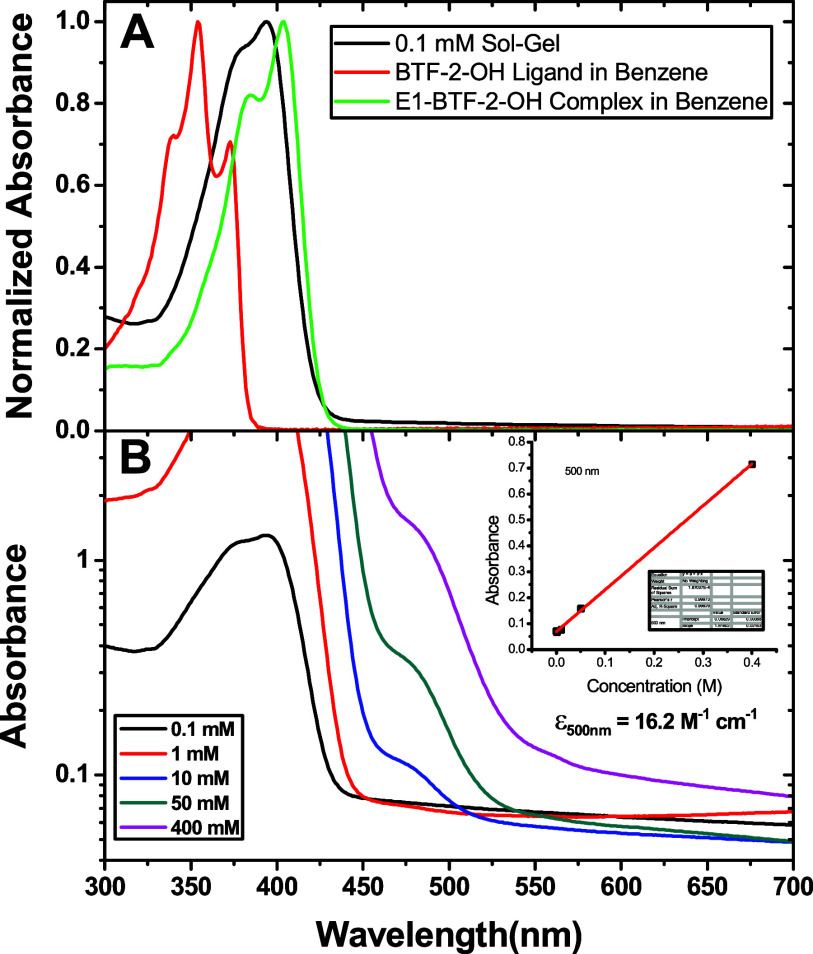
(A) Ground-state absorption spectra of the 0.1 mM E1-BTF-2-OH complex dissolved in the ormosil matrix, BTF-2-OH ligand, and E1-BTF-2-OH complex dissolved in benzene. (B) Ground-state absorption spectra of the ormosil samples. The inset is a Beer’s law plot measuring the molar absorption coefficient at 500 nm.

Ground-state absorption spectra of all concentrations of the glasses referenced against air are shown in Figure 2B. A new feature first appears at a 10 mM concentration with a 485 nm absorption maximum. The molar absorption coefficient is 16.2 M^–1^ cm^–1^ at 500 nm. We previously measured absorption spectra of platinum acetylides PE1, PE2, and PE3.^15^ In these compounds, there is a weak transition (ε < 10 M^–1^ cm^–1^) appearing as a shoulder red-shifted from the S_0_ → S_1_ band assigned as the S_0_ → T_1_ transition. Due to the heavy atom effect in the platinum complexes on spin–orbit coupling, the weak spin-forbidden S_0_ → T_1_ transition is made possible. This direct transition is also likely in E1-BTF-2-OH and would be observed only at high concentrations. In the ormosil samples, we observe two absorption bands for the 10 mM to 400 mM samples with an S_0_ → S_1_ band at 394 nm and a shoulder at 485 nm. The latter band we suspect is probably some combination of an S_0_ → T_1_ transition and dye aggregation at higher concentrations. The measured molar absorption coefficient is significantly larger than what was observed in the platinum acetylide complexes, suggesting some additional phenomena are occurring that scale with concentration.

Steady-state emission spectra (Figure 3A–D, Table 1) obtained by excitation at 375 and 485 nm with inner filter effects minimized by using front-face geometry illumination.^49^ There are still some reabsorption effects that must be considered in the data analysis. The emission spectra obtained from 375 nm excitation show a red shift from 437 nm at 0.1 mM to 573 nm at 400 mM. The intensity obtained from 375 nm excitation decreases with concentration, while the intensity obtained from 485 nm excitation increases with concentration.

**Figure 3:**
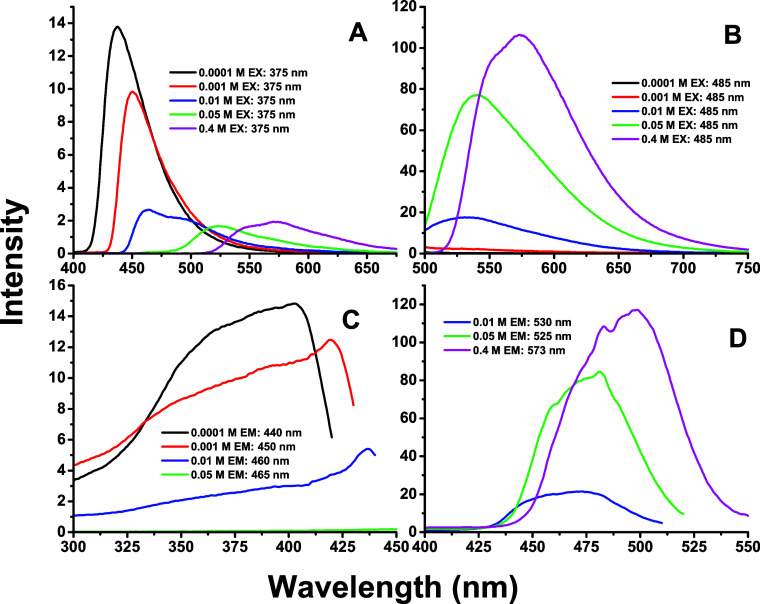
Steady-state emission and excitation spectra of the ormosil samples. The spectra were measured with front-face geometry to minimize inner filter effects. A: Emission spectra obtained from exciting the samples at 375 nm. B: Emission spectra obtained from exciting the samples at 485 nm. C: Excitation spectra obtained from monitoring the emission intensity in the range 440–465 nm. D: Excitation spectra obtained from monitoring the emission intensity in the range 530–573 nm.

**Table 1:** Ground-State Absorbance and Emission Data for Ormosil Glasses

	Abs_max_ ^a^	Fl_max_ ^b^	Fl_max_ ^c^	τ_s_ @ 440 nm^d^	τ_s_ @ Fl_max_	τ_s_ @ 570 nm
0.1 mM	394 nm	437 (13.7)	—	<50	<50	76; 733
1 mM	—	451 (9.8)	∼531 (0.48)	<50	<50	61; 538
10 mM	—	464 (2.6)	531 (17.6)	<50	<50	72; 724
50 mM	—	523 (1.6)	540 (71.9)	<50	<50; 595	88; 796
400 mM	—	573 (1.9)	573 (103)	<50	103; 860	103; 860
Benzene	402 nm	419 nm	—	—	<50	—

a Absorption maximum (nm).

b Emission maximum (nm) of samples excited at 375 nm. Quantity in parentheses is emission intensity (a.u.).

c Emission maximum (nm) of samples excited at 485 nm. Quantity in parentheses is emission intensity (a.u.).

d Emission lifetime in ps following 375 nm excitation.

The excitation spectra monitoring emission at 440 nm mirror the chromophore–absorption spectrum up to 1 mM. At higher concentrations, the excitation spectra overlap with the absorption peak at 485 nm, making it difficult to collect meaningful data at higher concentrations. At 400 mM, the emission spectra overlap whether we excite at 375 or 485 nm.

We compared the emission spectra of the 400 mM sample excited at 485 nm with the published phosphorescence spectrum of E1BTF in deoxygenated solution^19^ (Figure 4, bottom panel). We estimate the intrinsic lifetime of the emitting T_1_ → S_0_ transition by fitting the excitation spectrum obtained from the 400 mM sample (Figure 4, top panel) to a Gaussian function and obtain an oscillator strength *f* = 1.66 × 10^–4^.^50^ The intrinsic emissive lifetime of this weak band is estimated to be 14.5 μs. The 400 mM ormosil sample shows a broadband, less structured emission that overlaps with the solution phosphorescence spectrum.

**Figure 4:**
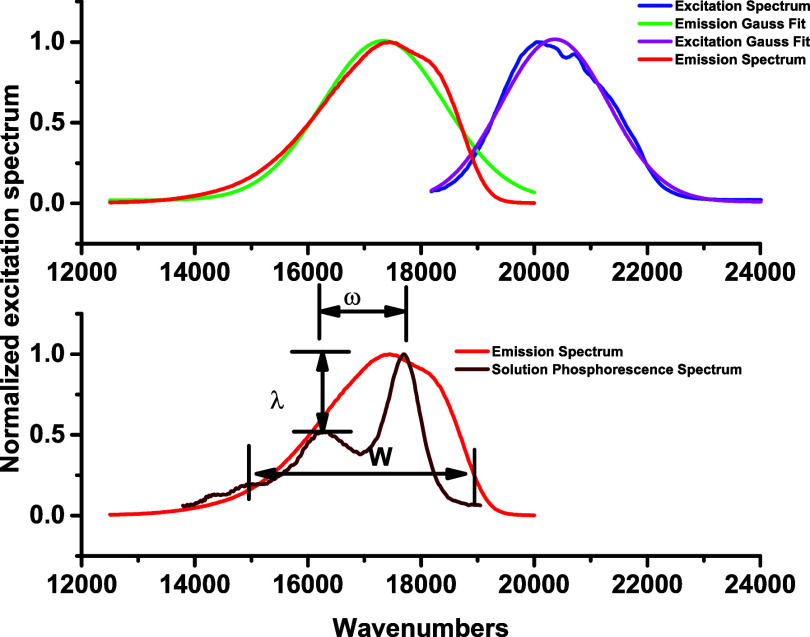
Top: Emission and excitation spectra for 0.4 M sample. Each spectrum was fitted to a single Gaussian curve. Fitting parameters are *V*_em_ = 17,336 cm^–1^, σ_em_ = 1,083 cm^–1^, *V*_ex_ = 20,367 cm^–1^, and σ_ex_ = 946 cm^–1^. Bottom: Emission spectrum for 0.4 M sample compared with published E1-BTF in benzene. Parameters are Huang–Rhys parameter for the 0–1 band: λ = 0.72; Exciton bandwidth: *W* = 4,000–7,000 cm^–1^, vibration frequency of 0–1 band: ω = 1,400 cm^–1^.

Emission lifetime data obtained from the TCSPC measurements are shown in Table 1. The samples were excited at 375 nm with a 70 ps pulse, and the emission was monitored at three wavelengths (440 nm to monitor fluorescence, wavelength of maximum intensity, and 570 nm to monitor phosphorescence). No rise and decay kinetics of emission consistent with excimer formation were observed. At 440 nm, the emission is solely from a monomer based on the peak of the lowest concentration sample. The 440 nm emission is within the instrument response function of ∼50 ps. In both the wavelength of maximum intensity and the 570 nm data, there is a longer-lived emission showing dual exponential decay appearing in the 50 mM sample and higher concentration samples. The longer lifetimes at 570 nm are around 1 ns, much less than the calculated intrinsic emission lifetime of 14.5 μs.

As the concentration varies, we observe several differences. Due to the mirror-like behavior of the emission to the absorption up to 1 mM, we assume that the environment of the E1-BTF-2-OH within the glass is similar to that observed in solution. From 10 mM up to 400 mM, a red shifting in the emission is observed and attributed to an increase in phosphorescence at the higher concentrations. Also, due to the high absorption in the higher concentration samples, there are likely large reabsorption effects of the S_1_–S_0_ emission. Interestingly, when comparing the emission data excited at 375 nm for the 10 mM and 400 mM samples, both indicate the presence of two peaks indicative of electronic relaxation to different vibrational levels in the ground state. For the data exciting at 485 nm, we also observe two peaks in the 400 mM sample only. The emission spectra provide insight into the shape of the S_0_ potential energy surface relative to the S_1_ potential energy surface, or in the case of phosphorescence, the T_1_ potential energy surface, suggesting that in the three higher concentration samples, there are environmental differences leading to shifts in the shape, forcing the ground state vibrational levels further apart, resulting in either distinct peaks or closer together, resulting in broadened emission data.

With increasing concentration, the emission intensity from 375 nm excitation decreases ∼7×, while that from 485 nm excitation increases ∼214× (Figure 3 A–D, Table 1), suggesting aggregation-induced-phosphorescence enhancement (AIPE).^28,51^ Systems having this behavior contain bonds and groups with free conformational rotation, including M–CC–, triphenylmethyl, and −C=C–. Upon aggregation, internal rotation is restricted, lowering the rate of radiationless decay to the ground state. A gold­(I) complex is nonemissive in one solvent but aggregates and becomes emissive upon changing solvent conformation.^52^ A series of binuclear organoplatinum­(II) complexes with foldable oligo­(orthophenyleneethynylene) groups exhibit enhanced phosphorescence upon solvent-induced aggregation.^53^ The rotational potential energy surfaces of a series of platinum acetylides containing phenyl–CC–Pt–CC–phenyl structural features exhibit free rotation about the phenyl–CC–Pt group in the ground and T_1_ state.^14^ Aggregation would inhibit this free rotation, decreasing the rate of radiationless decay and increasing the quantum yield.

### Excited-State Lifetimes

The excited-state lifetimes obtained from TA and MTA due to excitation at 355, 400, and 485 nm are listed in Table 2. The decay data were fit to a multiexponential model, and some representative data are shown in the Supporting Information. The initial fast decay τ_1_ ∼ 2 ps is due to intramolecular vibrational relaxation from upper electronic levels and host reorganization. Solvent rotational diffusion with a 2.4 ps lifetime has been reported in platinum acetylide complexes.^54^ The second decay τ_2_ ∼ 25 ps is likely intersystem crossing (ISC) to the triplet state and correlates well with the data collected using TCSPC (Table 1) where the lifetimes were all less than the instrument response function of 50 ps. Lifetimes τ_3_ and τ_4_ correspond to the decay of the triplet state to the ground state. The τ_3_ and τ_4_ values decrease with increasing concentration due to ground-state quenching, adsorption to ormosil sites, and aggregation. The 0.1 mM sample has one triplet decay lifetime (τ_3_) while the higher concentration samples have two triplet decay lifetimes. The 1 mM sample has the largest τ_1_, τ_2_, and τ_4_ values of all the samples, which could result from excimer formation.

**Table 2:** S_1_–S_*n*_ and T_1_–T_*n*_ Properties of E1-BTF Sol–Gel Glasses

	S_1_–S_*n*_ ^a^	τ_1_ (ps)	τ_2_ (ps)	T_1_–T_*n*_ ^b^	τ_3_	τ_4_	T_1_–T_*n*_ ^c^	τ_5_
0.1 mM	625 nm	2.1 ± 0.5	23.6 ± 6.8	595 nm	2.2 ± 0.4 μs	—	—	—
1 mM	624 nm	4.1 ± 2.2	43 ± 15	595 nm	770 ± 457 ns	10.6 ± 2.8 μs	—	—
10 mM	623 nm	2.6 ± 0.5	29.7 ± 6.7	620 nm	227 ± 44 ns	735 ± 37 ns	530 nm	500 ± 86 ns
50 mM	618 nm	2.2 ± 0.3	27.4 ± 8.7	640 nm	184 ± 8 ns	624 ±26 ns	530 nm	587 ± 102 ns
400 mM	615 nm	1.7 ± 0.5	24.4 ± 15.9	670 nm	43 ±13 ns	295 ± 47 ns	540 nm	5.7 ± 0.6 μs
Benzene^d^ (deoxygenated)	640 nm	—	23 ± 10	670 nm	126 μs	—	—	—

a Femtosecond excitation at 400 nm with lifetimes τ_1_ and τ_2_.

b Nanosecond flash photolysis excitation at 355 nm with lifetimes τ_3_ and τ_4_.

c Nanosecond flash photolysis excitation at 485 nm with lifetime τ_5_.

d Published value.^11^ The lifetime in oxygenated benzene is 318 ns.

Figure 5 is a plot of triplet decay rate constants *k*_3_, *k*_4_, and *k*_5_ as a function of concentration. The ormosil host behaves as a porous, nonhomogeneous environment with multiple dye-binding sites affecting the triplet decay rate constants, for example, pore surfaces vs interior. The triplet decay rate constant *k*_3_ = 4.5 × 10^5^ s^–1^ for the 0.1 mM sample is ∼100× larger than that measured for deoxygenated benzene, PMMA, and epoxy hosts at a low concentration (*k* ∼ 10^3^ s^–1^), suggesting the influence of dissolved oxygen in the porous matrix. For all concentrations, the triplet decay rate constant *k*_3_ (=1/τ_3_) includes the effect of oxygen quenching and adsorption to host sites (eq 1).

1 $$k_{3}=k_{0}+k_{q}P_{O_{2}}+K_{d}{[\mathrm{Dye}]}^{1/2}$$

**Figure 5:**
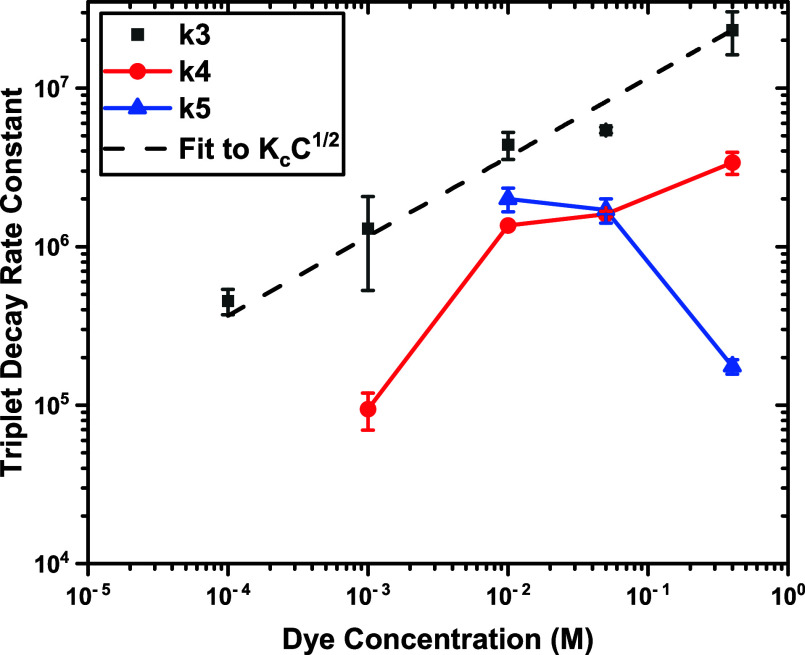
Triplet-state decay rate constants plotted from the data in Table 2. The *k*_3_ = 1/τ_3_ were fitted to the Freundlich equation. *k*_4_ and *k*_5_ values correspond to τ_4_ and τ_5_.

The terms include the intrinsic constant (*k*_0_), the effect of dissolved oxygen quenching $(k_{q}P_{O_{2}})$ , and ground-state quenching due to adsorption (*K*_d_[Dye]^1/2^). The last term is the empirical Freundlich equation describing the adsorption of the dye onto multiple sites having different energies of sorption.^55,56^

Atmospheric oxygen diffuses and dissolves in the ormosil.^57,58^ The dissolved oxygen concentration is proportional to oxygen partial pressure, host matrix solubility constant, and Henry’s law constant. The oxygen quenching rate constant (*k*) is proportional to the chromophore-oxygen encounter distance, diffusion coefficient, and chromophore excited-state lifetime. High-porosity materials like xerogels and ormosils have *k*_q_ values (3–2,300 Pa^–1^s^–1^), while low-porosity materials like polystyrene, Teflon, and silicone rubber have *k*_q_ values (3–80 Pa^–1^ s^–1^).^59^ At sea level, the partial pressure of atmospheric oxygen is 21,223 Pa. For a triplet decay rate constant of the 0.1 mM sample, *k*_3_ = 4.5 × 10^5^ s^–1^ and *k*_q_ = 21.2 Pa^–1^ s^–1^, which are within published ranges. At low concentrations, E1-BTF-2-OH exhibits phosphorescence in both the PMMA^10^ and epoxy^11^ hosts but is quenched in the ormosil host, probably due to higher porosity and adsorption effects.^60^ The phosphorescence of platinum octaethyl porphyrin (PtOEP) dissolved in solid hosts illustrates host-oxygen-quenching effects. The phosphorescence intensity of PtOEP dissolved in a high-oxygen-permeability ormosil host decreases 40-fold as the percentage of oxygen increases to 100%, while in a low-oxygen-permeability polymer host, it decreases only 2- to 3-fold.^61^

The triplet decay rate constant *k*_4_ (=1/τ_4_) has more complex behavior than *k*_3_. The slow triplet decay seen in the 1 mM sample is associated with charge transfer effects observed in TA measurements (Figure 7). We excited the 10, 50, and 400 mM samples at 485 nm directly into the T_1_ state rate to obtain rate constant *k*_5_. Comparing these rate constants shows 10 mM: *k*_5_ (485 nm excitation) > *k*_4_ (355 nm excitation); 50 mM: *k*_5_ (485 nm excitation) ∼ *k*_4_ (355 nm excitation); 400 mM: *k*_5_ (485 nm excitation) < *k*_4_ (355 nm excitation), suggesting different dye populations whether excited at 355 or 485 nm.

Published single-molecule spectroscopy studies show a multimodal distribution of dye environments in ormosil systems.^62^ Ormosils have density ranging from 1.2 to 1.4 g/mL,^63^ giving [Si] ∼ 7 M. For all the samples, [Dye] ≪ [Si], so the doped ormosil structure is like undoped ormosil. An average ormosil has 50% porosity, pore diameter 3.5 nm, and pore volume 9.6 nm.^3,64^ It is likely that one dye population resides in the pores and the other in the nonporous medium. From X-ray diffraction measurements on E1-BTF, we measured the crystal density to be 1.290 g/mL^19^ and the molecular volume to be 1.8 nm^3^. The estimated ratio of pore volume to molecular volume is ∼5, so there is sufficient core volume to accommodate a small aggregate.

Shown in Figure 6 are the overlaid triplet-state absorption spectra for the samples excited at 355 nm (A and B) and 485 nm (C) and probing from 400 to 900 nm. The spectra obtained immediately after excitation show a red shift in the peak maximum with the concentration (Figure 6A). In the lower panel, the spectra of the 0.1 and 1 mM samples measured greater than 7 triplet lifetimes after excitation show no change in band shape (Figure 6B). The spectra of the 10 and 50 mM samples show a peak at ∼500 nm superimposed on a broad band, interpreted as resulting from exciton interactions and aggregation effects. We obtained triplet-state spectra by excitation into the T_1_ state at 485 nm (Figure 6C). For the 1 and 0.1 mM samples, no signal was observed. The 10, 50, and 400 mM samples exhibited a band in the range 530–540 nm superimposed on a broad absorption band.

**Figure 6:**
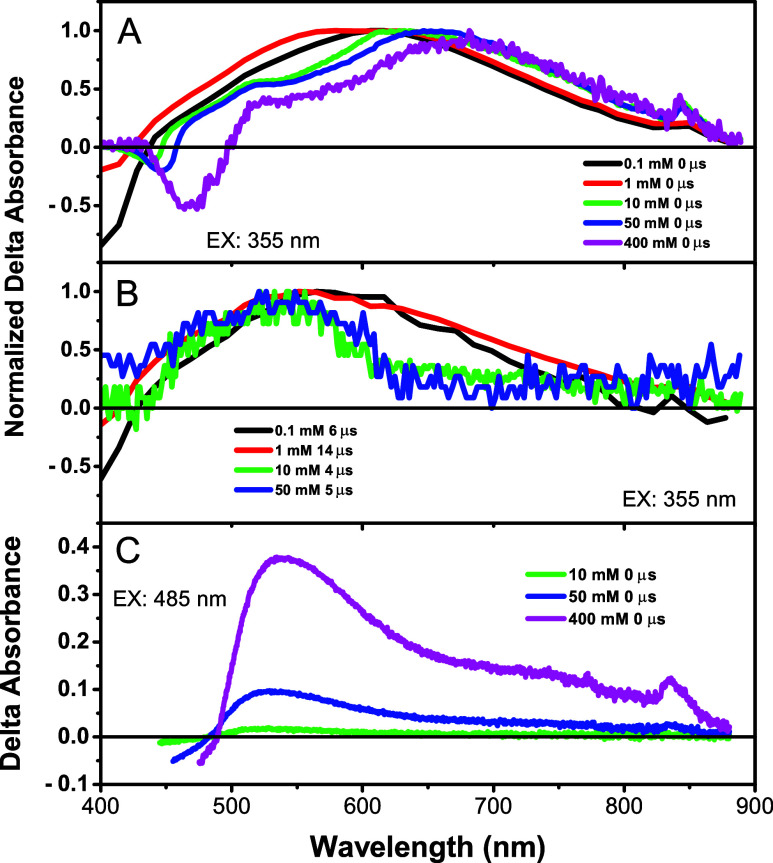
Triplet-state absorption spectra obtained from the excitation of the samples at 355 nm. (A) Data immediately following the pulse at time zero and (B) data taken at >4 μs. Due to high optical density, the signal was too weak to measure a spectrum of the 400 mM sample at long times. (C) Triplet-state absorption spectra obtained by excitation of the samples at 485 nm and taken immediately following the pulse.

### Excited-State Exciton Coupling

We probed transitions from the |S_1_⟩ state to the |S_2_⟩ state by exciting the sample with a 400 nm, 50 fs pulse and probing with a white-light continuum. Figure 7 shows TA spectra obtained from 0 ps to immediately after ISC followed by longer-time spectra. There are two absorption bands appearing after excitation from the |S_1_⟩ state into higher states plus a negative-ground-state depletion band. The presence of two bands suggests excited-state symmetry breaking (ESSB) in this system. Studies of ESSB in other systems show that the chromophore is symmetric and quadrupolar in nonpolar solvents, but symmetry breaking occurs in polar solvents.^38,39^ We previously studied one- and two-photon spectra of platinum acetylides in terms of symmetry breaking.^12,42^ We found that the excited-state dipole moment of symmetry-broken conformations calculated by TDDFT most agreed with excited-state dipole moments measured by two-photon spectroscopy. Molecular conformations arising from ESSB in the current ormosil system are a function of dye concentration, adsorption to the matrix, and aggregation.

**Figure 7:**
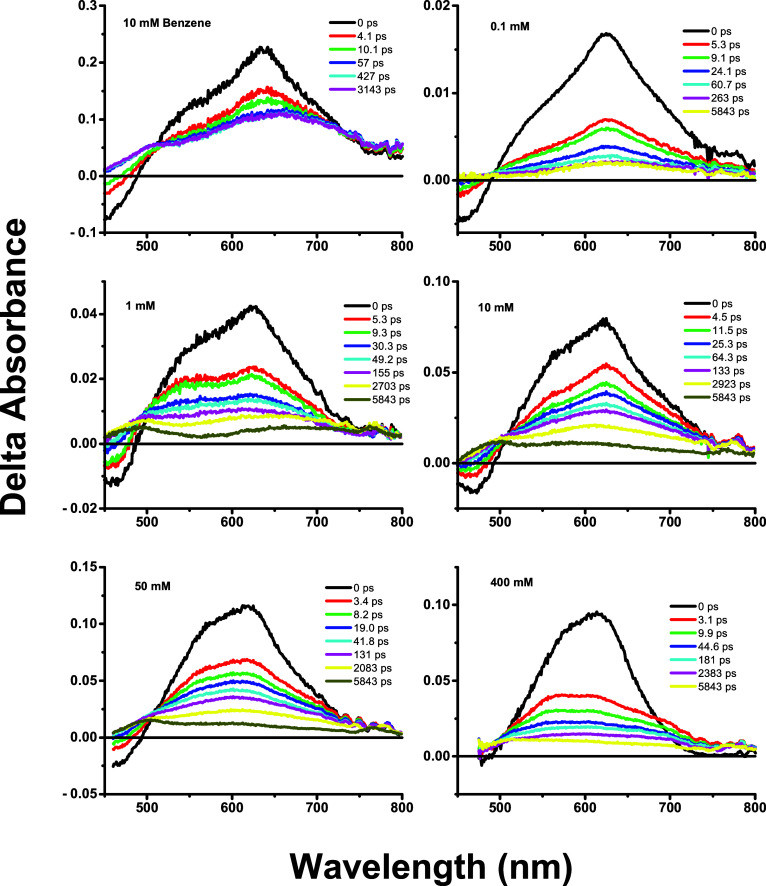
Ultrafast transient absorption spectra of the samples following 400 nm of excitation. Times are in the range ≤τ_2_ which include vibrational relaxation, host reorganization, and intersystem crossing. Longer times are in the range τ_2_ < *t* < ∞ which include excimer formation and appearance of long-lifetime spectra.

We analyzed the TA spectra as intramolecular–Frenkel–exciton interactions between the two ligand-localized states.^22,65,66^ The ground-state wave function (|*S*_0_⟩) is a product of ligand mixed with Pt d-orbital terms.

2 $$\left|S_{0}\rangle =\right|g_{1}g_{2}\rangle$$

The excited state |E_1_⟩ is visualized as the interaction of two locally excited ligands.

3 $$\left|S_{1}\rangle =\right|{\mathrm{LE}}_{1}^{1}\rangle ,\mathrm{or},|{\mathrm{LE}}_{2}^{1}\rangle$$

The subscript and superscript denote the ligand and state, respectively. The pump pulse causes the |S_0_⟩ → |S_1_⟩ transition. The |S_1_⟩ state is delocalized over both ligands. The transient absorption spectrum immediately after excitation (0 ps) describes the |S_1_⟩ → |S_2_⟩ transition before the dye excited state undergoes dephasing and energy exchange with the surrounding medium. The |S_2_⟩ state has contributions from both ligands

4 $$\left|S_{2}\rangle =\right|{\mathrm{LE}}_{1}^{2}\rangle ,\mathrm{or},|{\mathrm{LE}}_{2}^{2}\rangle$$

The interaction energy (*J*_c_) between excited-state dipoles μ_1_ and μ_2_ in a medium with dielectric constant ε, separated by the shortest center-to-center distance (*r*), angles between the transition moments, and the angle between the transition moment vectors (α) is given by the expression

5 $$J_{c}=\frac{\mu^{2}}{4\pi \varepsilon r^{3}}\cos\left(\alpha \right)$$

The |S_2_⟩ state splits into upper and lower states having energies

6 $$E^{\prime }=E-J_{c}-V,\mathrm{and},E^{\prime \prime }=E+J_{c}+V$$

and wave functions

7 $$|{S_{2}\rangle }^{\prime }=\frac{1}{\sqrt{2}}\left(\left|{\mathrm{LE}}_{1}^{2}\rangle +\right|{\mathrm{LE}}_{2}^{2}\rangle \right)$$

and

8 $$|{S_{2}\rangle }^{\prime \prime }=\frac{1}{\sqrt{2}}\left(\left|{\mathrm{LE}}_{1}^{2}\rangle -\right|{\mathrm{LE}}_{2}^{2}\rangle \right)$$

The function |S_2_⟩″ is described as “in-phase”, while the function |S_2_⟩″ is described as “out-of-phase”. The splitting between the two peaks is

9 $$\Delta E=E^{\prime \prime }-E^{\prime }=2\left|J_{c}\right|+2V$$

The quantity *V* describes intramolecular splitting due to mechanisms not involving dipole–dipole interactions. The dimer transition dipole moments follow the relation

10 $${\mathrm{\mu ⃗}}^{\prime }=\frac{1}{\sqrt{2}}\left({\mathrm{\mu ⃗}}_{1}+{\mathrm{\mu ⃗}}_{2}\right),\mathrm{and},{\mathrm{\mu ⃗}}^{\prime \prime }=\frac{1}{\sqrt{2}}\left({\mathrm{\mu ⃗}}_{1}-{\mathrm{\mu ⃗}}_{2}\right)$$

The sum of the transition dipoles forms parallel and perpendicular components: $\mu_{∥}=\sqrt{2}\mu \cos\left(\frac{\alpha }{2}\right)$ and $\mu_{⊥}=\sqrt{2}\mu \sin\left(\frac{\alpha }{2}\right)$ . Measurement of the area ratio of the two bands enables calculation of the angle between the transition dipoles.

11 $$\frac{{\mathrm{Area}}^{\prime \prime }}{{\mathrm{Area}}^{\prime }}=\frac{f^{\prime \prime }}{f^{\prime }}=\frac{\nu^{\prime }}{\nu^{\prime \prime }}tan^{2}\left(\frac{\alpha }{2}\right)$$

When α = 0 deg and *J*_c_ > 0, *E*′ > *E*″, the in-phase excited state is the upper state, and the out-of-phase excited state is the lower state and the excited state spectrum will show one band. When α = 90 deg, *J*_c_ = 0, and the two dipoles are independent and the excited state spectrum will show two bands having the same intensity and splitting 2*V*. The quantities α and Δ­(*E*) measure molecular conformation, aggregation, and environment changes over time.

We used the TA data on the various samples to learn about structure changes as a function of concentration and time after 400 nm excitation. The fitted data are shown in the Supporting Information. Table 3 gives measurements of *E*′, *E*″, Area′, and Area″ at *t* = 0 ps, after intersystem crossing (*t* = 133–427 ps) and longer times (*t* = 3,000–6,000 ps). The quantity Δ­(*E*) measures changes in the coulomb (*J*_c_) and other (*V*) interactions resulting from intersystem crossing and relaxation processes. When α = 0 deg, there will be one band at energy *E*′, interpreted as a “planar” conformation. As α increases from 0 to 90 deg, the intensity of the *E*″ band will increase. When α = 90 deg, two bands with equal intensities will appear, interpreted as a “nonplanar” conformation. The quantity Δ­(α)_*t*_ = α_*t*_ – α_0_ measures time-resolved changes relative to the 0 ps spectrum resulting from vibrational cooling, local environment reorganization, intersystem crossing, aggregation, and triplet state relaxation. Shown in Figure 8 are plots Δ­(*E*) and α as functions of time and concentration. The times shown are 0 ps (α_0_, Δ­(*E*)_0_), after intersystem crossing (α_isc_, Δ­(*E*)_isc_), and long times after the pump pulse (α_∞_, Δ­(*E*)_∞_).

**Table 3:**
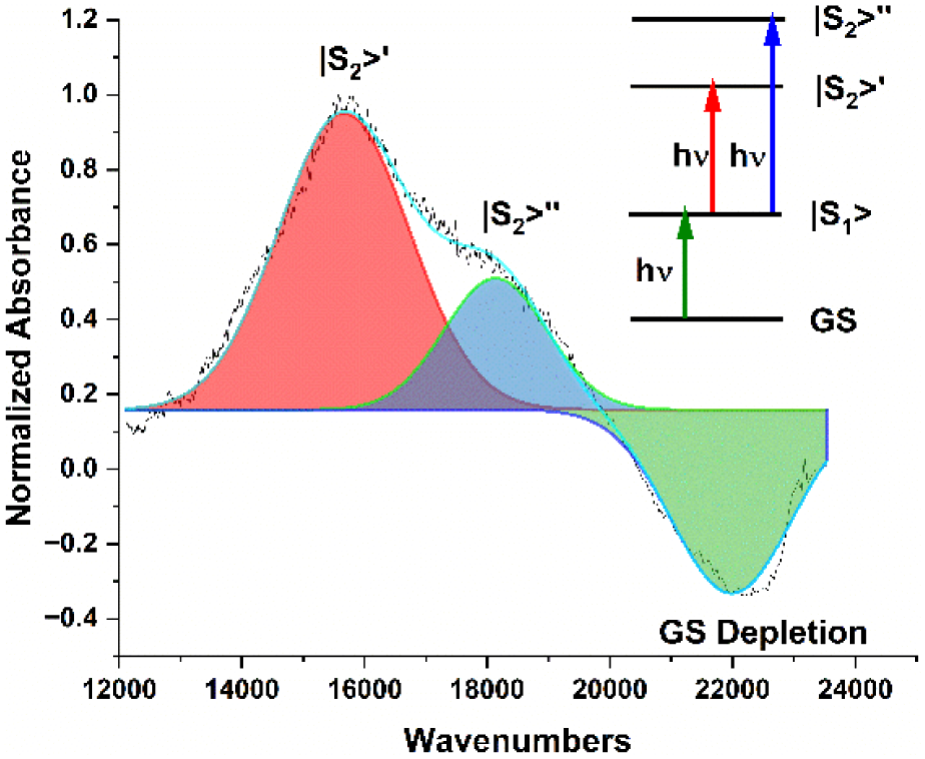
Ultrafast Spectra Band Fit Summary^a^

System	Time^a^	*E*′^b^	*E*″	Δ(*E*)^c^	*A*″/*A*′^d^	α^e^	Δ(α)_isc_ ^f^	Δ(α)_∞_ ^g^
0.1 mM	0	15,926	18,303	2,377	0.272	58.5	—	—
0.1 mM	263	15,697	19,361	3,664	0.090	36.9	–21.5	—
0.1 mM	5,843	15,827	19,361	3,534	0.090	36.8	—	–21.6
1 mM	0	16,026	18,403	2,377	0.472	72.7	—	—
1 mM	155	16,293	19,854	3,561	0.393	69.3	–3.38	—
1 mM	5,843	14,692	20,286	5,594	0.576	83.4	—	10.7
10 mM	0	16,017	18,059	2,042	0.413	68.6	—	—
10 mM	133	16,197	19,253	3,056	0.162	47.4	–21.1	—
10 mM	5,843	16,768	20,049	3,281	0.511	76.0	—	7.41
50 mM	0	16,144	18,132	1,988	0.424	69.2	—	—
50 mM	131	16,418	19,343	2,925	0.218	53.8	–15.4	—
50 mM	5,843	16,866	19,902	3,036	0.678	83.6	—	14.3
400 mM	0	16,359	18,375	2,016	0.328	62.5	—	—
400 mM	181	16,272	18,929	2,657	0.293	60.6	–1.91	—
400 mM	5,843	16,952	19,459	2,507	0.535	76.1	—	13.6
10 mM Benzene	0	15,663	18,135	2,472	0.348	64.8	—	—
10 mM Benzene	427	15,421	19,108	3,687	0.124	42.9	–21.9	—
10 mM Benzene	3,143	15,466	19,164	3,698	0.127	43.3	—	–21.4

a Time (ps). The TA spectrum was fit to Gaussian functions as shown in the Supporting Information. The *t* = 0 ps spectrum was fit assuming *w*′ = 2,138 cm^–1^ and *w*″ = 1,809 cm^–1^. At times after intersystem crossing, *w*′ = 3,213 cm^–1^ and *w*″ = 1,809 cm^–1^.

b State energy (cm^–1^).

c Δ­(*E*) = *E″* – *E*′.

d Area ratio.

e Calculated angle (deg) between transition dipoles μ′ and μ″.

f Δ­(α)_isc_ is the change in angle after intersystem crossing referenced to time = 0 ps.

g Δ­(α)_∞_ is the change in angle after large times referenced to time = 0 ps.

**Figure 8:**
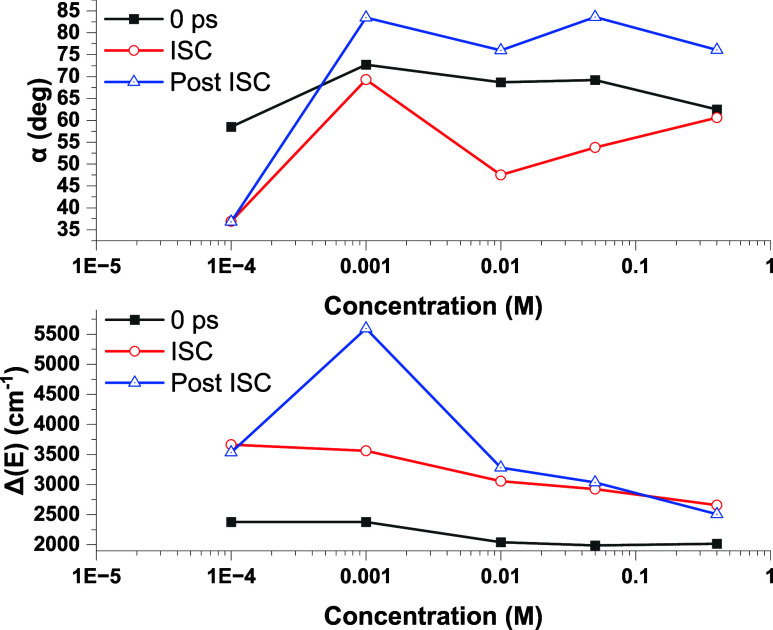
Summary of results of the Gaussian fit of TA spectra as a function of concentration and time after excitation. The times shown are 0 ps after excitation, after intersystem crossing (*t* = 181–263 ps), and long times (*t* = 5,843 ps). The top panel shows the calculated angle α between the transition dipoles as a function of concentration and time, while the bottom panel shows the measured energy gap between the upper and lower Gaussian bands.

### Behavior of 0.1 mM Sample

The ground-state absorption spectrum of the 0.1 mM sample is like that of E1-BTF-2OH dissolved in benzene. There is no evidence of a shoulder at 485 nm. There is blue-shift and band broadening compared to the solution, but no changes in vibronic structure, indicating aggregate formation. Similarly, the emission and excitation spectra are similar to the solution spectra. The triplet-state spectra obtained from excitation at 355 nm show no band shape changes comparing 0 and 6 μs after excitation. The TA spectra collected in the time range 0–60 ps show an isosbestic point due to ISC, and the TA spectra in the time range 78–5,843 ps show no change, suggesting a single species (Figure 7). Comparing TA spectra obtained from a solution in benzene showed similar short- and long-time behavior. We fit the TA spectra at 0, 263, and 5,843 ps. The energy gap between the states follows the trend Δ­(*E*)_0_ < Δ­(*E*)_isc_ ∼ Δ­(*E*)_∞_. The angle change referenced to 0 ps was negative, and Δ­(α)_isc_ ∼ Δ­(α)_∞_. The trends suggest that following excitation, there is intersystem crossing to a more planar dipole orientation and no changes in structure or composition at longer times. Table 3 also shows the data for E1-BTF-2-OH dissolved in benzene. As with the 0.1 mM sample dissolved in ormosil, the energy difference between the *E*′ and *E*″ states for the dye dissolved in benzene follows the trend Δ­(*E*)_0_ < Δ­(*E*)_isc_ ∼ Δ­(*E*)_∞_, the angle change Δ­(α) < 0 and Δ­(α)_isc_ ∼ Δ­(α)_∞_. It is concluded that the 0.1 mM sample dissolved in ormosil behaves like an oxygenated liquid solution, and there are no dye aggregation effects.

### Behavior of 1 mM Sample

In the 1 mM sample, there is no 485 nm aggregate band in the ground-state absorption spectrum. Emission and excitation spectra are red-shifted compared with the solution spectra. The triplet-state absorption spectrum of the 1 mM sample shows no change in band shape comparing the spectra 0 and 14 μs after excitation. The TA spectra in the time range 0–60 ps showed an isosbestic point resulting from ISC. We fit the TA spectra at 0, 155, and 5,843 ps. The energy gap between the *E*′ and *E*″ states follows the trend Δ­(*E*)_0_ < Δ­(*E*)_isc_ < Δ­(*E*)_∞_. The calculated angle change referenced to 0 ps follows the trend Δ­(α)_isc_ < Δ­(α)_∞_. The trends suggest that following excitation, there is ISC to the T_1_ state (Figure 7), and then Δ­(*E*) continues to increase due to charge transfer processes. Figure 9 is a plot of *E*′, *E*″, and absorbance at 625 nm over time. In the time range 20 ps < *t* < 100 ps, *E*″ increases with ISC. In the time range *t* = 100 to 5,000 ps, Δ­(*E*) continues to increase. The trend was not observed in any of the other samples and suggests excimer formation, where the excited states have radical cation (C) or anion (A) character.

12 $${\left|S_{2}\right\rangle }^{\prime }=a\left(\left|{\mathrm{LE}}_{1}^{2}\rangle +\right|{\mathrm{LE}}_{2}^{2}\rangle \right)+b\left(\left|C_{1}A_{2}\rangle +\right|C_{2}A_{1}\rangle \right)$$

13 $$|{S_{2}\rangle }^{\prime \prime }=a\left(\left|{\mathrm{LE}}_{1}^{2}\rangle -\right|{\mathrm{LE}}_{2}^{2}\rangle \right)-b\left(\left|C_{1}A_{2}\rangle +\right|C_{2}A_{1}\rangle \right)$$

**Figure 9:**
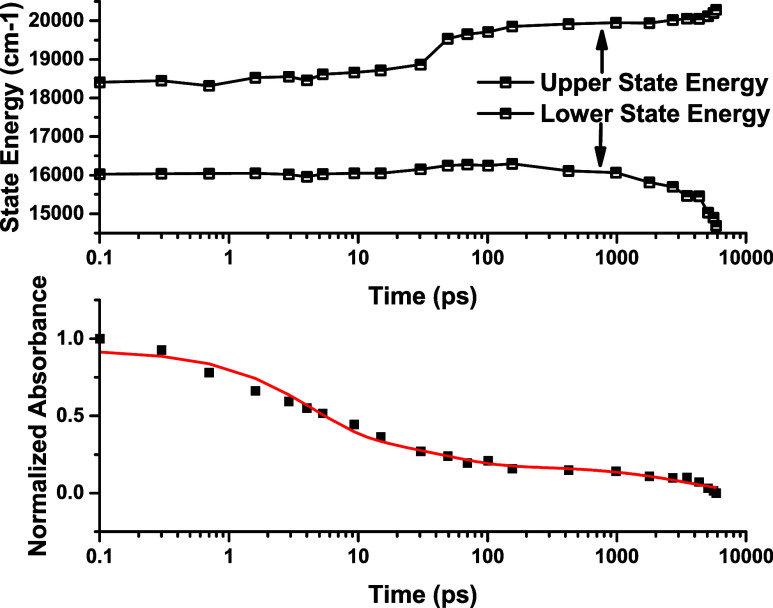
Bottom: Absorbance decay of the 1 mM sample was measured from ultrafast transient absorption spectra at 625 nm. Top: For each time, the TA spectrum was fitted to two Gaussians. The plot shows the energy of the upper (*E*″) and lower (*E*′) states.

Due to higher dye concentration, excimer formation occurs at 1 mM, although oxygen quenching suppresses phosphorescence. It is concluded that the 1 mM sample behaves as an excimer.

### Behavior of 10 mM, 50 mM, and 400 mM Samples

The ground-state spectra of the 10, 50, and 400 mM samples all show some degree of an S_0_ → T_1_ band at 485 nm. The triplet-state absorption spectra have a single broad band at 620–670 nm 0 μs after excitation. At long times after excitation, the triplet spectra have peaks near 500 nm. As observed in Figure 7 the TA spectra in the time range 0–100 ps show an isosbestic point due to ISC, while the TA spectra at longer times show bands at 500 and 625 nm. The energy gap between the |S_2_⟩″ and |S_2_⟩′″ states follows the trend Δ­(*E*)_0_ < Δ­(*E*)_isc_ ∼ Δ­(*E*)_∞_. The calculated angle change referenced to 0 ps follows the trend Δ­(α)_isc_ < 0 while Δ­(α)_∞_ > 0. At high concentrations, aggregation effects predominate. The two ligands of an E1-BTF-2-OH chromophore in an aggregated environment become “planar” after intersystem crossing, but as the triplet state relaxes, aggregation effects cause the conformation to become “nonplanar”. In previous work, we compared the triplet-state emission and excitation spectra of platinum acetylides indicating the triplet-state potential energy surface has a narrow molecular conformation distribution compared to the ground state which has a broad molecular conformation distribution.^14^

Figure 6C shows the triplet state absorption spectra of the 10, 50, and 400 mM samples appearing immediately after 485 nm excitation. Curve fitting was performed on these samples (figures shown in Supporting Information) and is summarized in Table 4 and compares them to *E*′, *E*″, and Δ­(*E*)_∞_ calculated from the same samples from TA data. The trends are *E*′(485 nm ex) ∼ *E*′(TA)_∞_; *E*″(485 nm ex) < *E*″(TA)_∞_; Area″/Area′(485 nm ex) ∼ Area″/Area′(TA)_∞_; α­(485 nm ex) ∼ α­(TA)_∞_. The difference in *E″* values results from two different pathways to create the T_1_ state. In the TA experiment, the sample is excited with a 50 fs pulse into the S_1_ state, followed by intersystem crossing and formation of a relaxed T_1_ state. In the MTA experiment, the ground state is excited directly into the T_1_ state by a 485 nm pulse. It is concluded that the 10, 40, and 400 mM samples are aggregated.

**Table 4:** Spectroscopic Parameters from 485 nm Excitation

Sample	*E*′^a^	*E*″^a^	Δ(*E*)^b^	Area″/Area′	α^c^
10 mM	15,531	18,403	2,872	0.920	92.5
50 mM	16,222	18,475	2,253	0.640	81.0
400 mM	16,789	18,339	1,550	0.477	71.6
Average	16,181 ± 514	18,406 ± 55	2,253 ± 514	0.679 ± 0.183	81.7 ± 8.5
Average from TA data	16,868 ± 67	19,803 ± 251	2,941 ± 323	0.575 ± 0.073	78.5 ± 3.6

a State energy (cm^–1^).

b Δ­(*E*) = *E*″ – *E*′.

c Calculated angle (deg) between transition dipoles μ′ and μ″.

Practical use of ormosils at high dye concentrations requires suppression of aggregation. In the current work, the dye aggregates at some point during fabrication.^46^ Fabrication involves acidic hydrolysis and condensation of a mixture of MTEOS and the dye dissolved in THF, followed by gel aging and drying to form a xerogel. During condensation, the added chromophore precipitates if the solvent is removed too quickly, leaving an insufficient number of cross-links with the gel. At the end of the condensation, the chromophores are completely cross-linked with the matrix, and the gel could be shaped and dried.

Hansen solubility parameters, δ (MPa^1/2^),^67^ describe solubility in terms of dispersion (D), polarity (P), and hydrogen bonding (H) parameters: (δ_D_, δ_P_, δ_H_).^68^ A solute has a solubility sphere with a radius of *R*_0_.

14 $$R_{0}^{2}=4{\left(\delta_{D}\right)}^{2}+{\left(\delta_{P}\right)}^{2}+{\left(\delta_{H}\right)}^{2}$$

Solubility is a function of the solubility parameter differences between the solute and the host.

15 $$R_{a}^{2}=4{\left(\Delta \delta_{D}\right)}^{2}+{\left(\Delta \delta_{P}\right)}^{2}+{\left(\Delta \delta_{H}\right)}^{2}$$

The ratio RED = *R*_a_/*R*_o_ describes the solubility behavior. When RED < 1, the solute dissolves. As RED approaches 1, solubility decreases and when RED > 1, the solute aggregates. The three cohesive energy terms (δ_D_, δ_P_, δ_H_) for THF are (16.8, 5.7, 8.0) and for MTEOS are (14.1, 3.9, 4.1).^69^ For silica, the terms are (15.2, 12.0, 13.0).^70^ E1-BTF-2OH is soluble (RED < 1) in THF and MTEOS but aggregates (RED > 1) in the ormosil as δ_P_ (THF, MTEOS) < δ_P_ (silica) and δ_H_ (THF, MTEOS) < δ_H_ (silica), (RED > 1). During hydrolysis and condensation, the mixture becomes more polar, leading to dye aggregation, aggregation-induced luminescence and other concentration effects. Increasing the dye’s δ_P_ and δ_H_ values as well as decreasing the ormosil’s δ_P_ and δ_H_ values will increase solubility.

The short triplet-state lifetimes compared with PMMA and epoxy systems result from oxygen quenching. Oxygen barrier materials, including nanocomposites, polymer–polymer multilayers, cross-linked polymers and crystalline polymers have been developed.^71^ Applying an oxygen barrier coating on the disk surface would decrease *k*_q_ and increase the triplet lifetime.

## Conclusions

We prepared a series of ormosil glasses containing nonlinear chromophore E1-BTF-2-OH at a wide concentration range. There is evidence for dissolved oxygen in the samples due to shorter triplet decay rate constants and lack of phosphorescence compared to the dye dissolved in PMMA and epoxy hosts. At higher concentrations, dye aggregation effects appear in the emission and excited-state absorption spectra. We used exciton theory to study interligand interactions associated with probing from the |S_1_⟩ state. The measured parameters α and Δ­(*E*) are a function of excited-state relaxation and dye concentration. These parameters are useful for understanding excited-state behavior including dye conformation changes, host reorganization, intersystem crossing, charge transfer, and aggregation.

## Supplementary Material



## Notes

Representative decay data fit to a multiexponential model, fitted data, TA spectrum fit to Gaussian functions, and curve fitting data (PDF)
