# Supplementary material for: Aggregates and Excitons: Excited-State Behavior of Platinum–Acetylide Two-Photon Chromophore-Doped Ormosil Glasses
Source: J Phys Chem A. 2025 Oct 29;129(45):10425–36. doi: 10.1021/acs.jpca.5c05728 (PMC12980846; doi:10.1021/acs.jpca.5c05728)

Supplementary Information

Aggregates and excitons: excited-state behavior of platinum-acetylide two-photon-chromophore-doped ormosil glasses

Thomas M. Cooper[1], Jonathan E. Slagle[1], Douglas M. Krein[1,2] and Joy E. Haley[1]*

[1]Materials and Manufacturing Directorate

Air Force Research Laboratory

Wright-Patterson Air Force Base, OH 45433

[2]General Dynamics Information Technology

Dayton, OH 45420

Following Figure: Shows the triplet state decay kinetics with representative fits for 0.1 mM, 1 mM, 10 mM, 50 mM, and 400 mM. Data was averaged over multiple wavelengths and is shown in Table 2 with standard deviation.

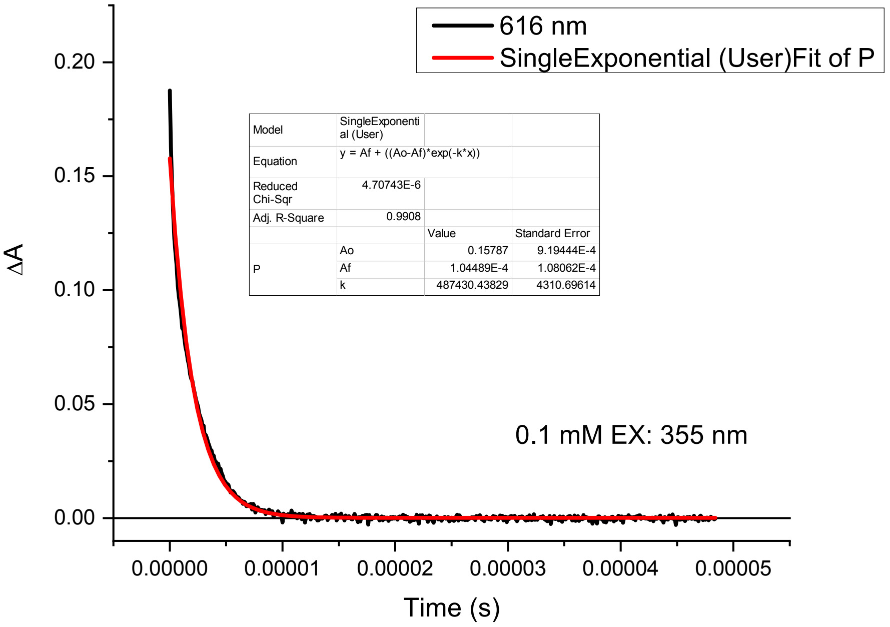

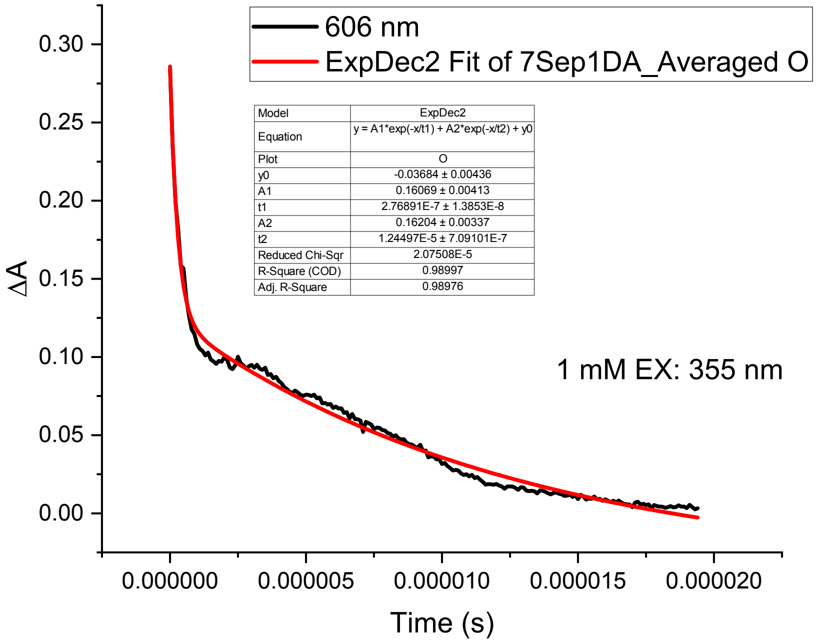

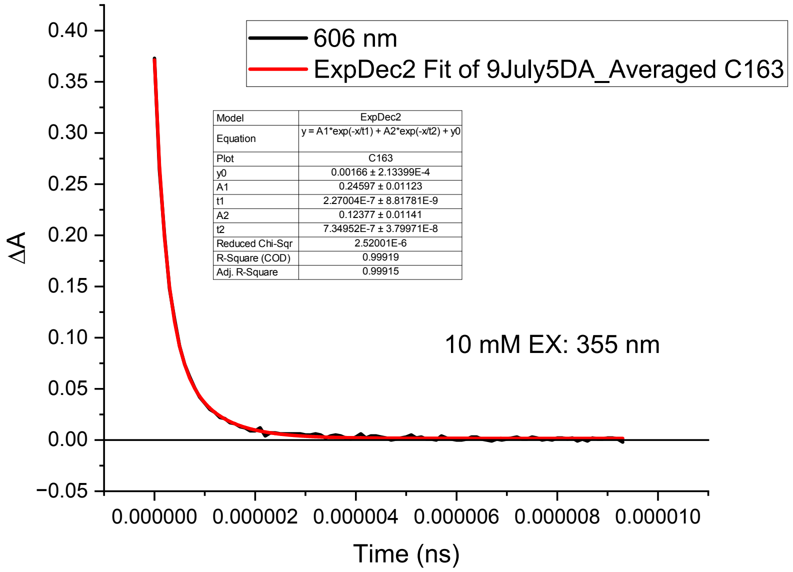
606 nm
ExpDec2 Fit of 9July5DA_Averaged C163
Model ExpDec2
Equation y = A1*exp(-x/t1) + A2*exp(-x/t2) + y0
Plot C163
y0 0.00166 ± 2.13399E-4
A1 0.24597 ± 0.01123
t1 2.27004E-7 ± 8.81781E-9
A2 0.12377 ± 0.01141
t2 7.34952E-7 ± 3.79971E-8
Reduced Chi-Sqr 2.52001E-6
R-Square (COD) 0.99919
Adj. R-Square 0.99915
10 mM EX: 355 nm
ΔA
0.40
0.35
0.30
0.25
0.20
0.15
0.10
0.05
0.00
−0.05
0.000000
0.000002
0.000004
0.000006
0.000008
0.000010
Time (ns)

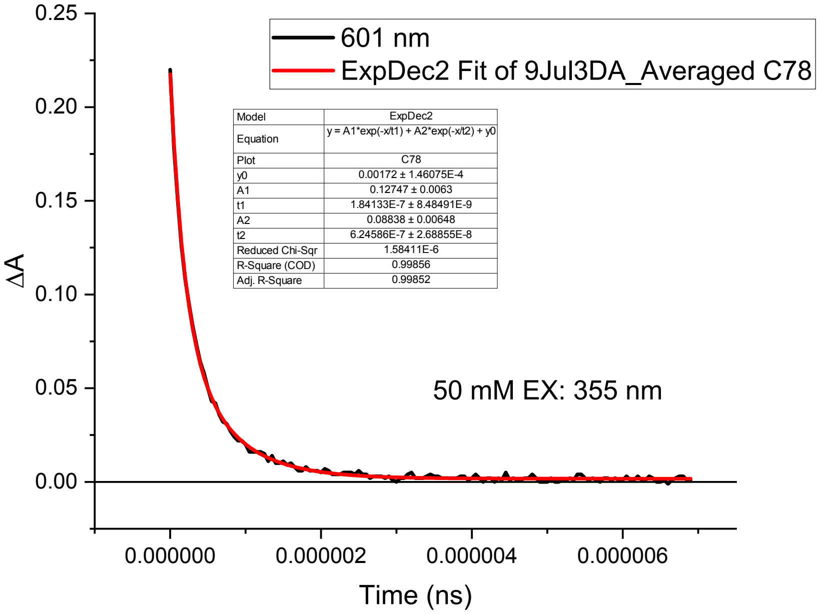
601 nm
ExpDec2 Fit of 9Jul3DA_Averaged C78
Model ExpDec2
Equation y = A1*exp(-x/t1) + A2*exp(-x/t2) + y0
Plot C78
y0 0.00172 ± 1.46075E-4
A1 0.12747 ± 0.0063
t1 1.84133E-7 ± 8.48491E-9
A2 0.08838 ± 0.00648
t2 6.24586E-7 ± 2.68855E-8
Reduced Chi-Sqr 1.58411E-6
R-Square (COD) 0.99856
Adj. R-Square 0.99852
50 mM EX: 355 nm
ΔA
0.25
0.20
0.15
0.10
0.05
0.00
0.000000
0.000002
0.000004
0.000006
Time (ns)

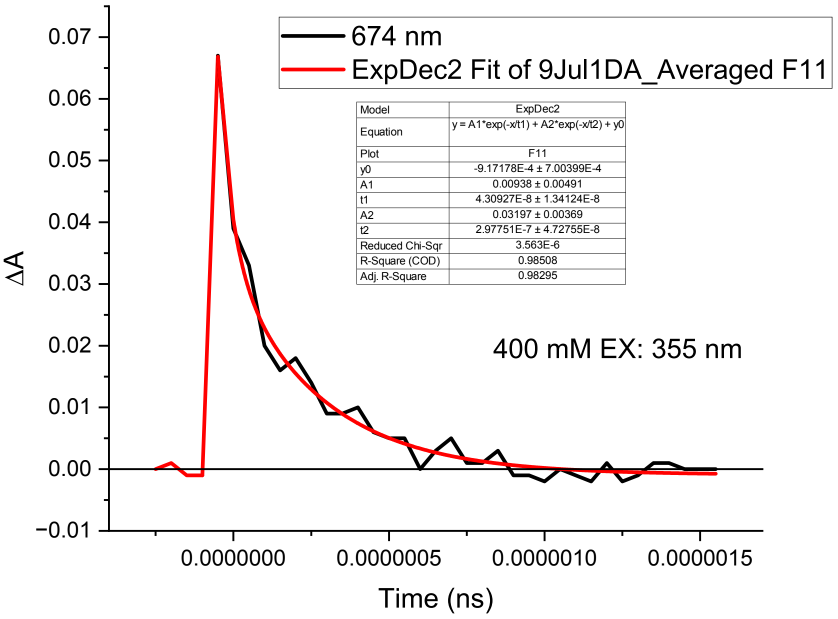

Model	ExpDec2
Equation	y = A1*exp(-x/t1) + A2*exp(-x/t2) + y0
Plot	F11
y0	-9.17178E-4 ± 7.00399E-4
A1	0.00938 ± 0.00491
t1	4.30927E-8 ± 1.34124E-8
A2	0.03197 ± 0.00369
t2	2.97751E-7 ± 4.72755E-8
Reduced Chi-Sqr	3.563E-6
R-Square (COD)	0.98508
Adj. R-Square	0.98295

Following Figures: Fit of spectral TA data at various times for each concentration set. The data has been captured in Table 3

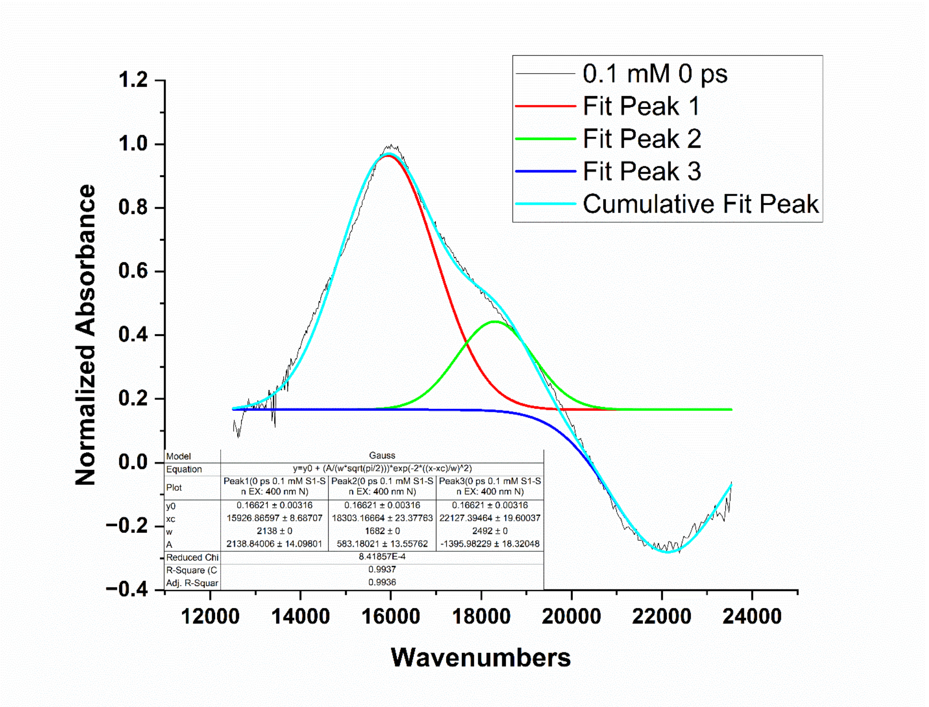

Model	Gauss		
Equation	y=y0 + (A/(w*sqrt(pi/2)))*exp(-2*((x-xc)/w)^2)		
Plot	Peak1(0 ps 0.1 mM S1-Sn EX: 400 nm N)	Peak2(0 ps 0.1 mM S1-Sn EX: 400 nm N)	Peak3(0 ps 0.1 mM S1-Sn EX: 400 nm N)
y0	0.16621 ± 0.00316	0.16621 ± 0.00316	0.16621 ± 0.00316
xc	15926.86597 ± 8.68707	18303.16664 ± 23.37763	22127.39464 ± 19.60037
w	2138 ± 0	1682 ± 0	2492 ± 0
A	2138.84006 ± 14.09801	583.18021 ± 13.55762	-1395.98229 ± 18.32048
Reduced Chi	8.41857E-4		
R-Square (C	0.9937		
Adj. R-Squar	0.9936		

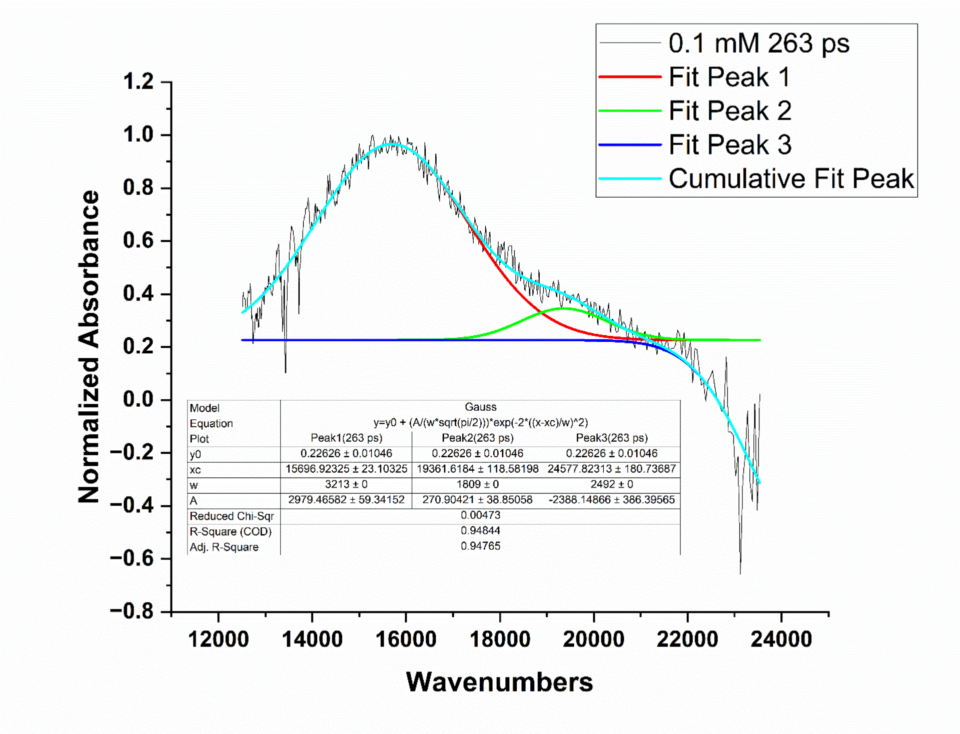

0.1 mM 263 ps
Fit Peak 1
Fit Peak 2
Fit Peak 3
Cumulative Fit Peak
Normalized Absorbance
1.2
1.0
0.8
0.6
0.4
0.2
0.0
−0.2
−0.4
−0.6
−0.8
12000
14000
16000
18000
20000
22000
24000
Wavenumbers
Model
Gauss
Equation
y=y0 + (A/(w*sqrt(pi/2)))*exp(-2*((x-xc)/w)^2)
Plot
Peak1(263 ps)
Peak2(263 ps)
Peak3(263 ps)
y0
0.22626 ± 0.01046
0.22626 ± 0.01046
0.22626 ± 0.01046
xc
15696.92325 ± 23.10325
19361.6184 ± 118.58198
24577.82313 ± 180.73687
w
3213 ± 0
1809 ± 0
2492 ± 0
A
2979.46582 ± 59.34152
270.90421 ± 38.85058
-2388.14866 ± 386.39565
Reduced Chi-Sqr
0.00473
R-Square (COD)
0.94844
Adj. R-Square
0.94765

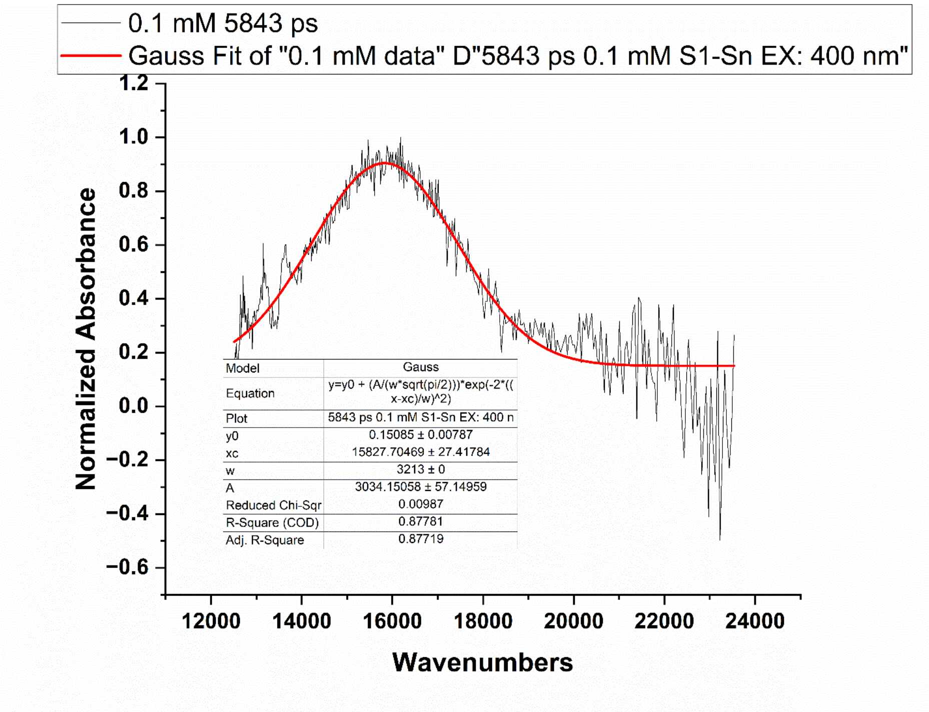

0.1 mM 5843 ps
Gauss Fit of "0.1 mM data" D"5843 ps 0.1 mM S1-Sn EX: 400 nm"
Normalized Absorbance
1.2
1.0
0.8
0.6
0.4
0.2
0.0
−0.2
−0.4
−0.6
12000
14000
16000
18000
20000
22000
24000
Wavenumbers
Model
Gauss
Equation
y=y0 + (A/(w*sqrt(pi/2)))*exp(-2*((x-xc)/w)^2)
Plot
5843 ps 0.1 mM S1-Sn EX: 400 n
y0
0.15085 ± 0.00787
xc
15827.70469 ± 27.41784
w
3213 ± 0
A
3034.15058 ± 57.14959
Reduced Chi-Sqr
0.00987
R-Square (COD)
0.87781
Adj. R-Square
0.87719

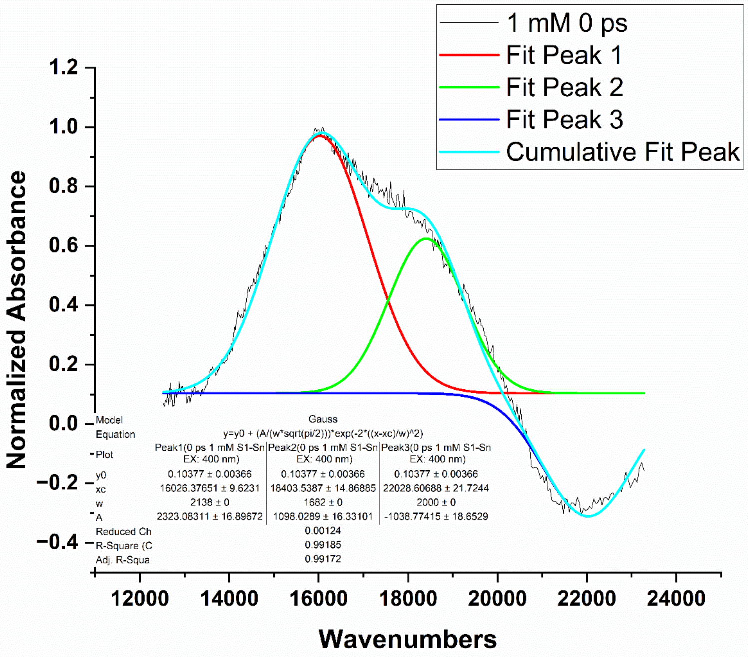

1 mM 0 ps
Fit Peak 1
Fit Peak 2
Fit Peak 3
Cumulative Fit Peak
Normalized Absorbance
Wavenumbers
1.2
1.0
0.8
0.6
0.4
0.2
0.0
−0.2
−0.4
12000
14000
16000
18000
20000
22000
24000
Model
Gauss
Equation
y=y0 + (A/(w*sqrt(pi/2)))*exp(-2*((x-xc)/w)^2)
Plot
Peak1(0 ps 1 mM S1-Sn EX: 400 nm)
Peak2(0 ps 1 mM S1-Sn EX: 400 nm)
Peak3(0 ps 1 mM S1-Sn EX: 400 nm)
y0
0.10377 ± 0.00366
0.10377 ± 0.00366
0.10377 ± 0.00366
xc
16026.37651 ± 9.6231
18403.5387 ± 14.86885
22028.60688 ± 21.7244
w
2138 ± 0
1682 ± 0
2000 ± 0
A
2323.08311 ± 16.89672
1098.0289 ± 16.33101
-1038.77415 ± 18.6529
Reduced Ch
0.00124
R-Square (C
0.99185
Adj. R-Squa
0.99172

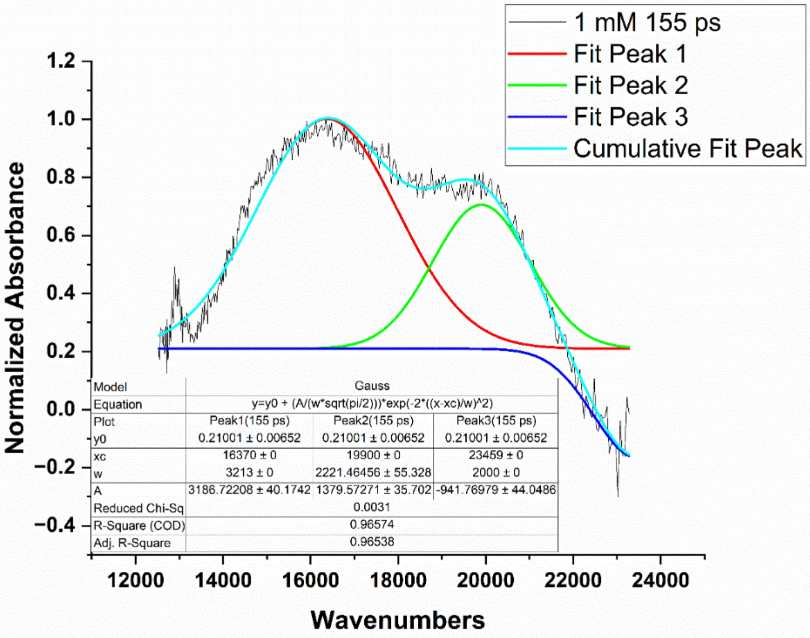

1 mM 155 ps
Fit Peak 1
Fit Peak 2
Fit Peak 3
Cumulative Fit Peak
Normalized Absorbance
Wavenumbers
1.2
1.0
0.8
0.6
0.4
0.2
0.0
−0.2
−0.4
12000
14000
16000
18000
20000
22000
24000
Model
Gauss
Equation
y=y0 + (A/(w*sqrt(pi/2)))*exp(-2*((x-xc)/w)^2)
Plot
Peak1(155 ps)
Peak2(155 ps)
Peak3(155 ps)
y0
0.21001 ± 0.00652
0.21001 ± 0.00652
0.21001 ± 0.00652
xc
16370 ± 0
19900 ± 0
23459 ± 0
w
3213 ± 0
2221.46456 ± 55.328
2000 ± 0
A
3186.72208 ± 40.1742
1379.57271 ± 35.702
-941.76979 ± 44.0486
Reduced Chi-Sq
0.0031
R-Square (COD)
0.96574
Adj. R-Square
0.96538

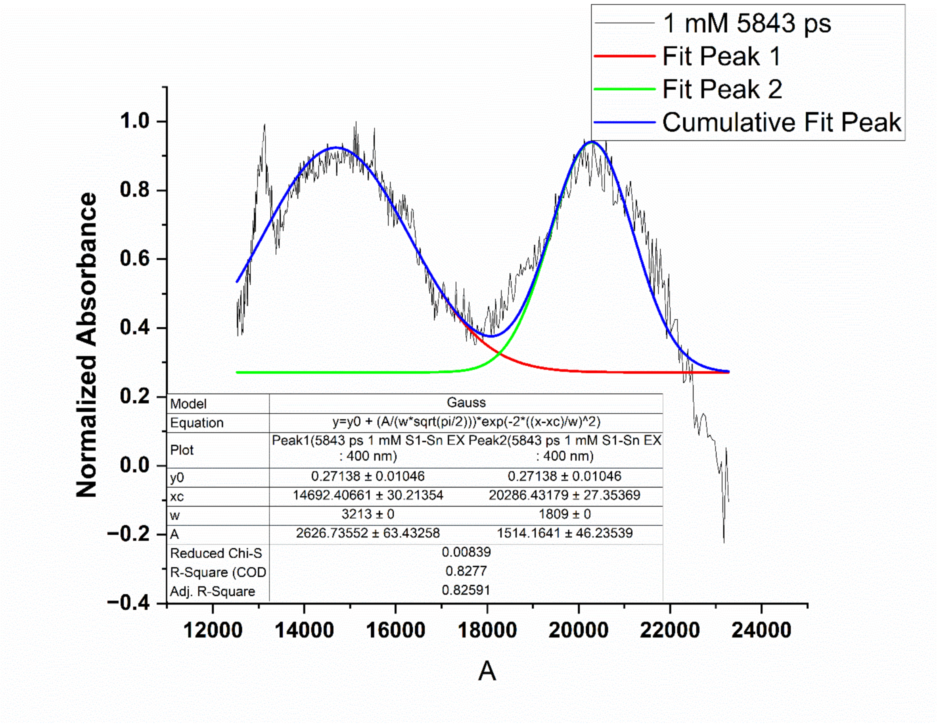
1 mM 5843 ps
Fit Peak 1
Fit Peak 2
Cumulative Fit Peak
Normalized Absorbance
1.0
0.8
0.6
0.4
0.2
0.0
−0.2
−0.4
Model | Gauss
Equation | y=y0 + (A/(w*sqrt(pi/2)))*exp(-2*((x-xc)/w)^2)
Plot | Peak1(5843 ps 1 mM S1-Sn EX : 400 nm) | Peak2(5843 ps 1 mM S1-Sn EX : 400 nm)
y0 | 0.27138 ± 0.01046 | 0.27138 ± 0.01046
xc | 14692.40661 ± 30.21354 | 20286.43179 ± 27.35369
w | 3213 ± 0 | 1809 ± 0
A | 2626.73552 ± 63.43258 | 1514.1641 ± 46.23539
Reduced Chi-S | 0.00839
R-Square (COD | 0.8277
Adj. R-Square | 0.82591
12000
14000
16000
18000
20000
22000
24000
A

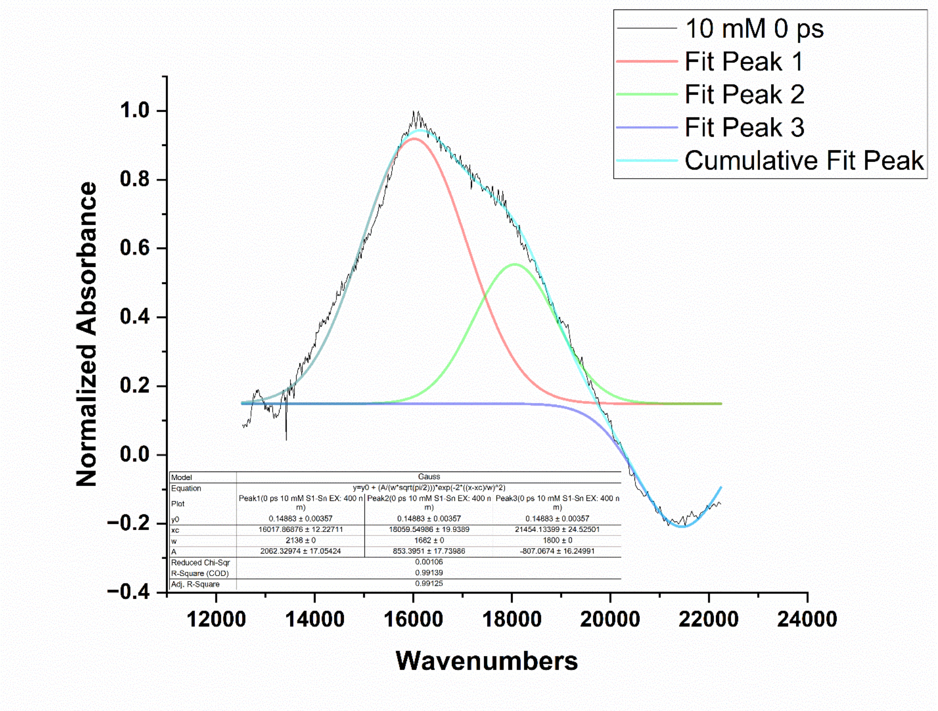
10 mM 0 ps
Fit Peak 1
Fit Peak 2
Fit Peak 3
Cumulative Fit Peak
Normalized Absorbance
1.0
0.8
0.6
0.4
0.2
0.0
−0.2
−0.4
Model | Gauss
Equation | y=y0 + (A/(w*sqrt(pi/2)))*exp(-2*((x-xc)/w)^2)
Plot | Peak1(0 ps 10 mM S1-Sn EX: 400 nm) | Peak2(0 ps 10 mM S1-Sn EX: 400 nm) | Peak3(0 ps 10 mM S1-Sn EX: 400 nm)
y0 | 0.14883 ± 0.00357 | 0.14883 ± 0.00357 | 0.14883 ± 0.00357
xc | 16017.86876 ± 12.22711 | 18059.54986 ± 19.9389 | 21454.13399 ± 24.52501
w | 2138 ± 0 | 1662 ± 0 | 1800 ± 0
A | 2062.32974 ± 17.05424 | 853.3951 ± 17.73986 | -807.0674 ± 16.24991
Reduced Chi-Sqr | 0.00106
R-Square (COD) | 0.99139
Adj. R-Square | 0.99125
12000
14000
16000
18000
20000
22000
24000
Wavenumbers

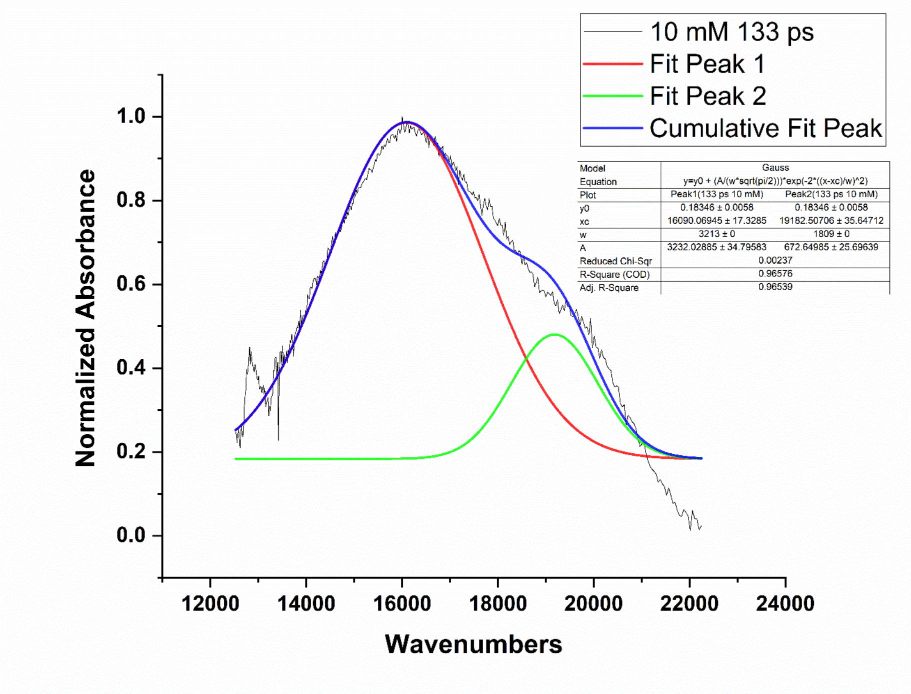
10 mM 133 ps
Fit Peak 1
Fit Peak 2
Cumulative Fit Peak
Model
Gauss
Equation
y=y0 + (A/(w*sqrt(pi/2)))*exp(-2*((x-xc)/w)^2)
Plot
Peak1(133 ps 10 mM)
Peak2(133 ps 10 mM)
y0
0.18346 ± 0.0058
0.18346 ± 0.0058
xc
16090.06945 ± 17.3285
19182.50706 ± 35.64712
w
3213 ± 0
1809 ± 0
A
3232.02885 ± 34.79583
672.64985 ± 25.69639
Reduced Chi-Sqr
0.00237
R-Square (COD)
0.96576
Adj. R-Square
0.96539
Normalized Absorbance
1.0
0.8
0.6
0.4
0.2
0.0
12000
14000
16000
18000
20000
22000
24000
Wavenumbers

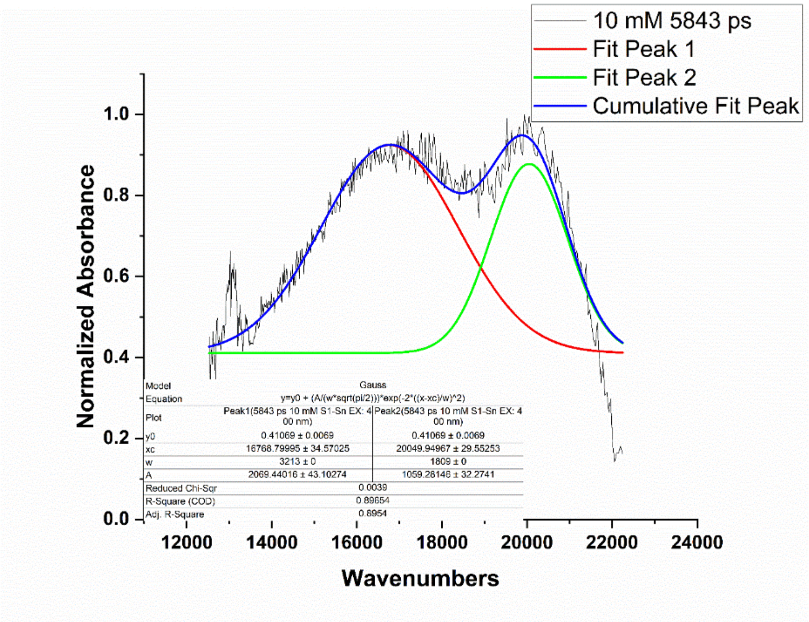
10 mM 5843 ps
Fit Peak 1
Fit Peak 2
Cumulative Fit Peak
Model
Gauss
Equation
y=y0 + (A/(w*sqrt(pi/2)))*exp(-2*((x-xc)/w)^2)
Plot
Peak1(5843 ps 10 mM S1-Sn EX: 400 nm)
Peak2(5843 ps 10 mM S1-Sn EX: 400 nm)
y0
0.41069 ± 0.0069
0.41069 ± 0.0069
xc
16768.79995 ± 34.57025
20049.94967 ± 29.55253
w
3213 ± 0
1809 ± 0
A
2069.44016 ± 43.10274
1059.28146 ± 32.2741
Reduced Chi-Sqr
0.0039
R-Square (COD)
0.89654
Adj. R-Square
0.8954
Normalized Absorbance
1.0
0.8
0.6
0.4
0.2
0.0
12000
14000
16000
18000
20000
22000
24000
Wavenumbers

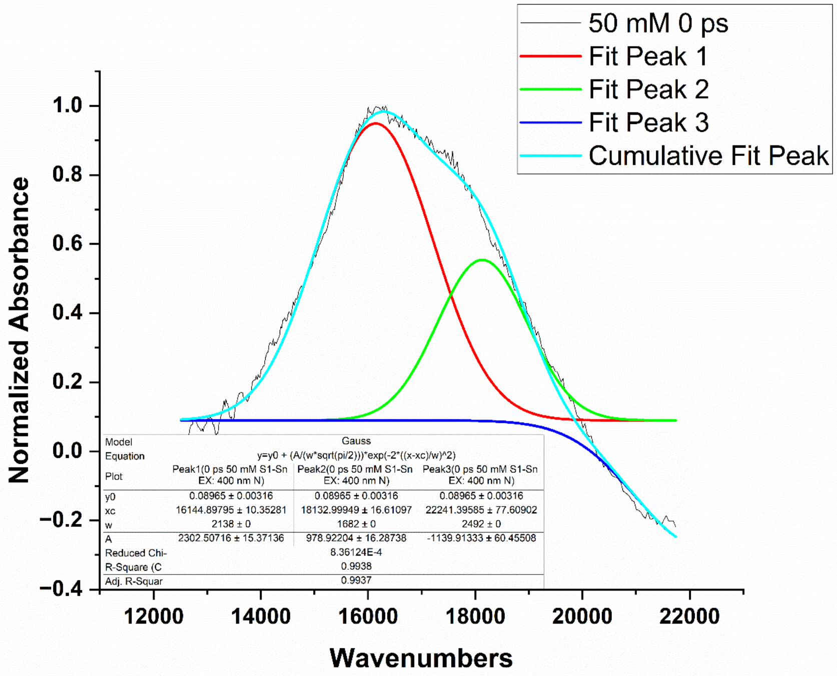
50 mM 0 ps
Fit Peak 1
Fit Peak 2
Fit Peak 3
Cumulative Fit Peak
Normalized Absorbance
Wavenumbers
1.0
0.8
0.6
0.4
0.2
0.0
−0.2
−0.4
12000
14000
16000
18000
20000
22000
Model Gauss
Equation y=y0 + (A/(w*sqrt(pi/2)))*exp(-2*((x-xc)/w)^2)
Plot Peak1(0 ps 50 mM S1-Sn EX: 400 nm N) Peak2(0 ps 50 mM S1-Sn EX: 400 nm N) Peak3(0 ps 50 mM S1-Sn EX: 400 nm N)
y0 0.08965 ± 0.00316 0.08965 ± 0.00316 0.08965 ± 0.00316
xc 16144.89795 ± 10.35281 18132.99949 ± 16.61097 22241.39585 ± 77.60902
w 2138 ± 0 1682 ± 0 2492 ± 0
A 2302.50716 ± 15.37136 978.92204 ± 16.28738 -1139.91333 ± 60.45508
Reduced Chi- 8.36124E-4
R-Square (C 0.9938
Adj. R-Squar 0.9937

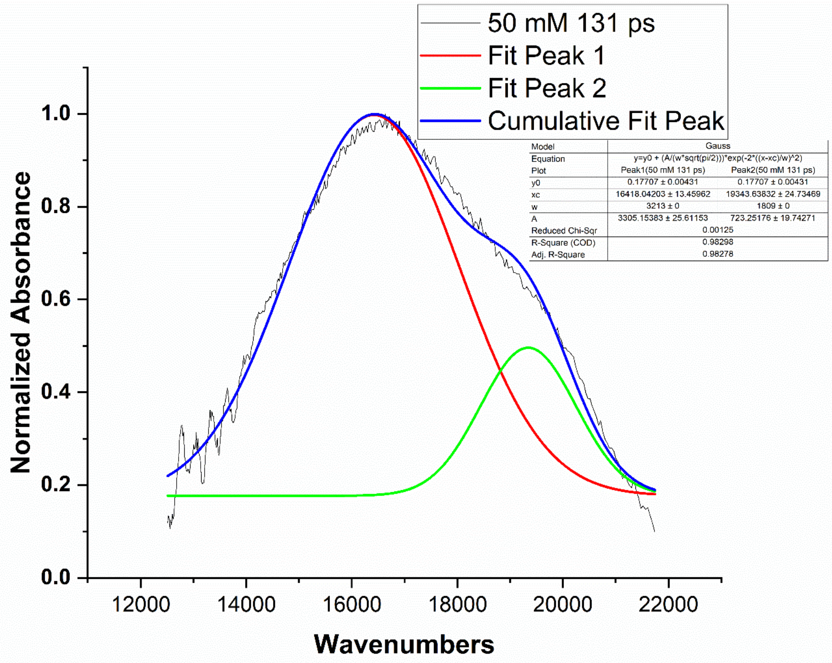
50 mM 131 ps
Fit Peak 1
Fit Peak 2
Cumulative Fit Peak
Normalized Absorbance
Wavenumbers
1.0
0.8
0.6
0.4
0.2
0.0
12000
14000
16000
18000
20000
22000
Model Gauss
Equation y=y0 + (A/(w*sqrt(pi/2)))*exp(-2*((x-xc)/w)^2)
Plot Peak1(50 mM 131 ps) Peak2(50 mM 131 ps)
y0 0.17707 ± 0.00431 0.17707 ± 0.00431
xc 16418.04203 ± 13.45962 19343.63832 ± 24.73469
w 3213 ± 0 1809 ± 0
A 3305.15383 ± 25.61153 723.25176 ± 19.74271
Reduced Chi-Sqr 0.00125
R-Square (COD) 0.98298
Adj. R-Square 0.98278

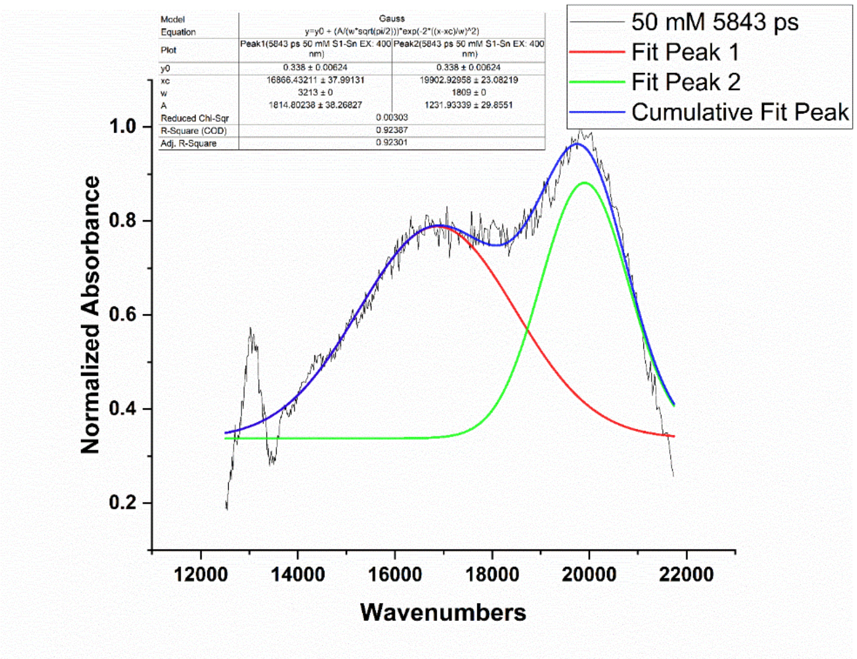
Model
Gauss
Equation
y=y0 + (A/(w*sqrt(pi/2)))*exp(-2*((x-xc)/w)^2)
Plot
Peak1(5843 ps 50 mM S1-Sn EX: 400 nm)
Peak2(5843 ps 50 mM S1-Sn EX: 400 nm)
y0
0.338 ± 0.00624
0.338 ± 0.00624
xc
16866.43211 ± 37.99131
19902.92958 ± 23.08219
w
3213 ± 0
1809 ± 0
A
1814.80238 ± 38.26827
1231.93339 ± 29.8551
Reduced Chi-Sqr
0.00303
R-Square (COD)
0.92387
Adj. R-Square
0.92301
50 mM 5843 ps
Fit Peak 1
Fit Peak 2
Cumulative Fit Peak
Normalized Absorbance
0.2
0.4
0.6
0.8
1.0
12000
14000
16000
18000
20000
22000
Wavenumbers

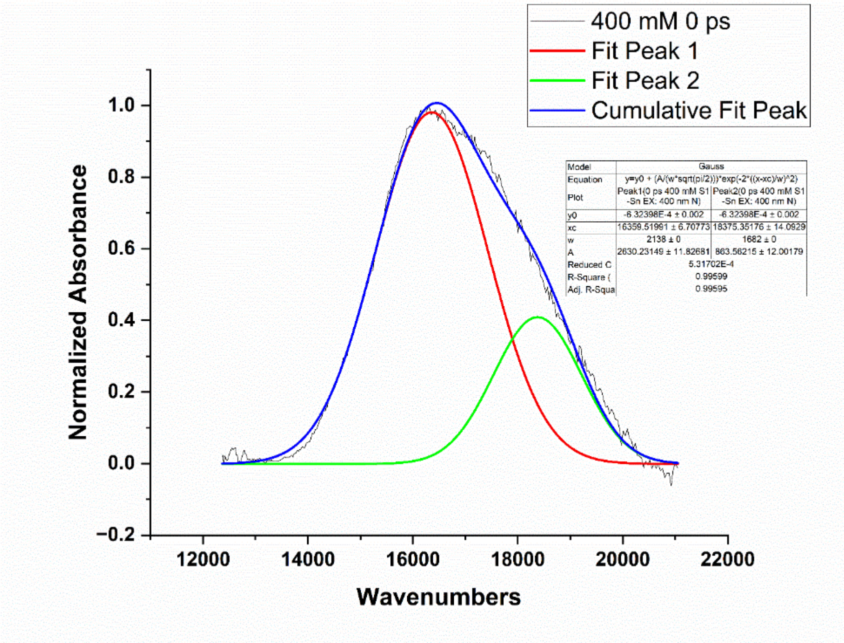
400 mM 0 ps
Fit Peak 1
Fit Peak 2
Cumulative Fit Peak
Model Gauss
Equation y=y0 + (A/(w*sqrt(pi/2)))*exp(-2*((x-xc)/w)^2)
Plot Peak1(0 ps 400 mM S1 -Sn EX: 400 nm N) Peak2(0 ps 400 mM S1 -Sn EX: 400 nm N)
y0 -6.32398E-4 ± 0.002 -6.32398E-4 ± 0.002
xc 16359.51991 ± 6.70773 18375.35176 ± 14.0929
w 2138 ± 0 1682 ± 0
A 2630.23149 ± 11.82681 863.56215 ± 12.00179
Reduced C 5.31702E-4
R-Square (0.99599
Adj. R-Squa 0.99595
Normalized Absorbance
Wavenumbers
1.0
0.8
0.6
0.4
0.2
0.0
−0.2
12000
14000
16000
18000
20000
22000

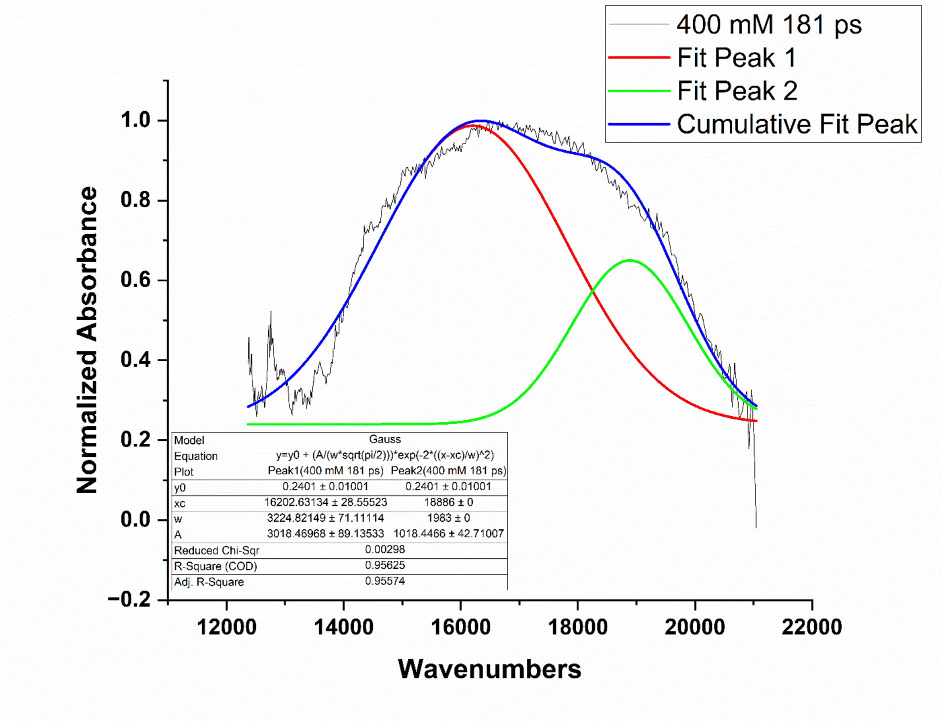
400 mM 181 ps
Fit Peak 1
Fit Peak 2
Cumulative Fit Peak
Model Gauss
Equation y=y0 + (A/(w*sqrt(pi/2)))*exp(-2*((x-xc)/w)^2)
Plot Peak1(400 mM 181 ps) Peak2(400 mM 181 ps)
y0 0.2401 ± 0.01001 0.2401 ± 0.01001
xc 16202.63134 ± 28.55523 18886 ± 0
w 3224.82149 ± 71.11114 1983 ± 0
A 3018.46968 ± 89.13533 1018.4466 ± 42.71007
Reduced Chi-Sqr 0.00298
R-Square (COD) 0.95625
Adj. R-Square 0.95574
Normalized Absorbance
Wavenumbers
1.0
0.8
0.6
0.4
0.2
0.0
−0.2
12000
14000
16000
18000
20000
22000

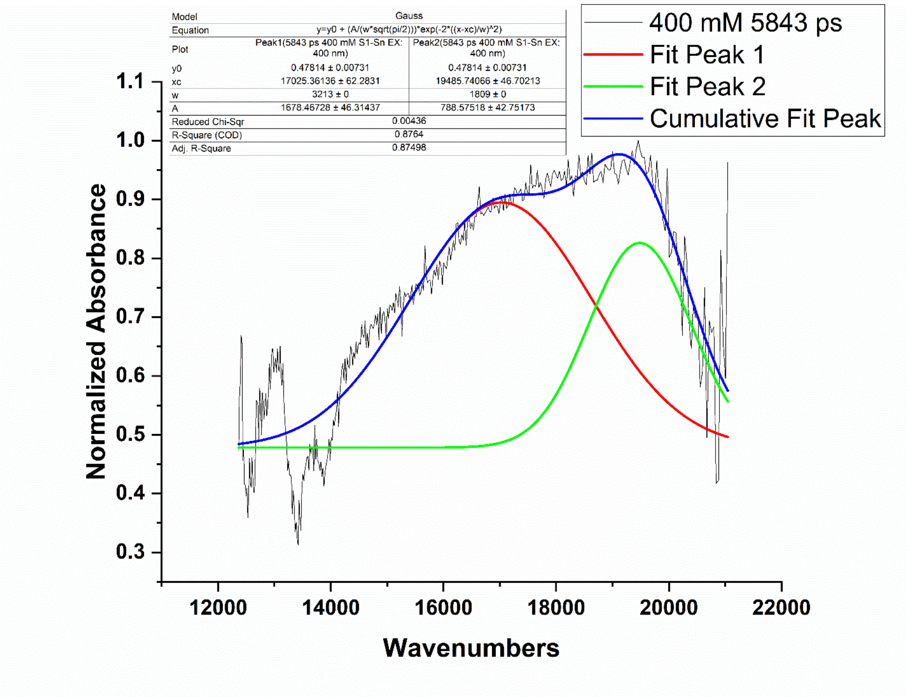
Model
Gauss
Equation
y=y0 + (A/(w*sqrt(pi/2)))*exp(-2*((x-xc)/w)^2)
Plot
Peak1(5843 ps 400 mM S1-Sn EX: 400 nm)
Peak2(5843 ps 400 mM S1-Sn EX: 400 nm)
y0
0.47814 ± 0.00731
0.47814 ± 0.00731
xc
17025.36136 ± 62.2831
19485.74066 ± 46.70213
w
3213 ± 0
1809 ± 0
A
1678.46728 ± 46.31437
788.57518 ± 42.75173
Reduced Chi-Sqr
0.00436
R-Square (COD)
0.8764
Adj. R-Square
0.87498
400 mM 5843 ps
Fit Peak 1
Fit Peak 2
Cumulative Fit Peak
Normalized Absorbance
1.1
1.0
0.9
0.8
0.7
0.6
0.5
0.4
0.3
12000
14000
16000
18000
20000
22000
Wavenumbers

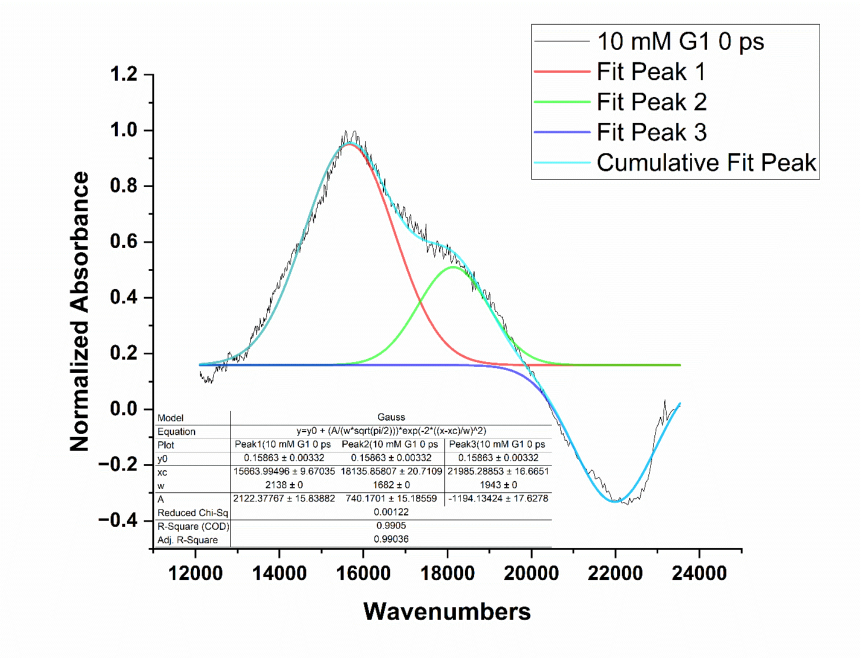
10 mM G1 0 ps
Fit Peak 1
Fit Peak 2
Fit Peak 3
Cumulative Fit Peak
Normalized Absorbance
1.2
1.0
0.8
0.6
0.4
0.2
0.0
−0.2
−0.4
Model
Gauss
Equation
y=y0 + (A/(w*sqrt(pi/2)))*exp(-2*((x-xc)/w)^2)
Plot
Peak1(10 mM G1 0 ps
Peak2(10 mM G1 0 ps
Peak3(10 mM G1 0 ps
y0
0.15863 ± 0.00332
0.15863 ± 0.00332
0.15863 ± 0.00332
xc
15663.99496 ± 9.67035
18135.85807 ± 20.7109
21985.28853 ± 16.6651
w
2138 ± 0
1682 ± 0
1943 ± 0
A
2122.37767 ± 15.83882
740.1701 ± 15.18559
-1194.13424 ± 17.6278
Reduced Chi-Sq
0.00122
R-Square (COD)
0.9905
Adj. R-Square
0.99036
12000
14000
16000
18000
20000
22000
24000
Wavenumbers

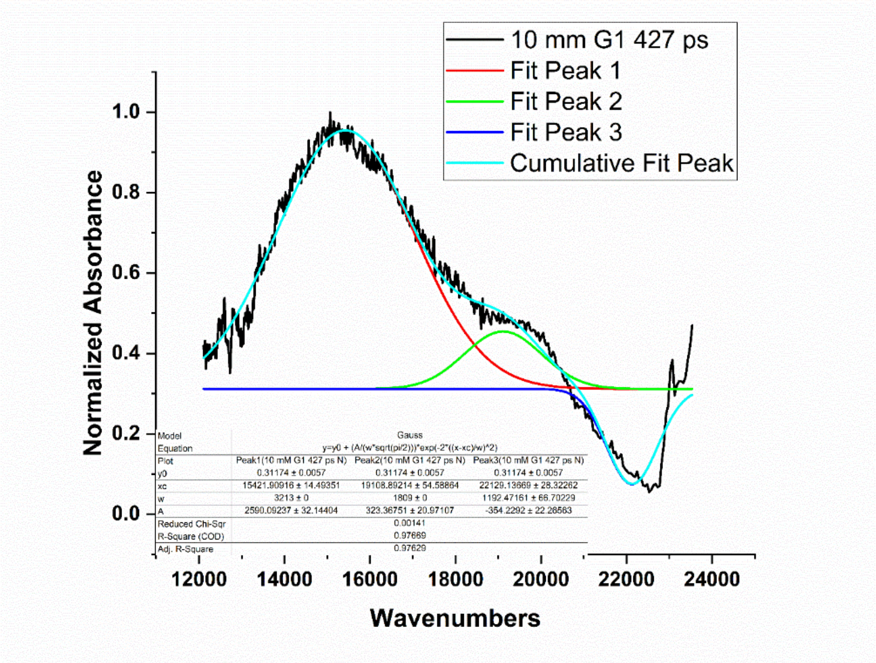
10 mm G1 427 ps
Fit Peak 1
Fit Peak 2
Fit Peak 3
Cumulative Fit Peak
Normalized Absorbance
Wavenumbers
Model
Gauss
Equation
y=y0 + (A/(w*sqrt(pi/2)))*exp(-2*((x-xc)/w)^2)
Plot
Peak1(10 mM G1 427 ps N)
Peak2(10 mM G1 427 ps N)
Peak3(10 mM G1 427 ps N)
y0
0.31174 ± 0.0057
0.31174 ± 0.0057
0.31174 ± 0.0057
xc
15421.90916 ± 14.49351
19108.89214 ± 54.58864
22129.13669 ± 28.32262
w
3213 ± 0
1809 ± 0
1192.47161 ± 66.70229
A
2590.09237 ± 32.14404
323.36751 ± 20.97107
-354.2292 ± 22.26563
Reduced Chi-Sqr
0.00141
R-Square (COD)
0.97669
Adj. R-Square
0.97629

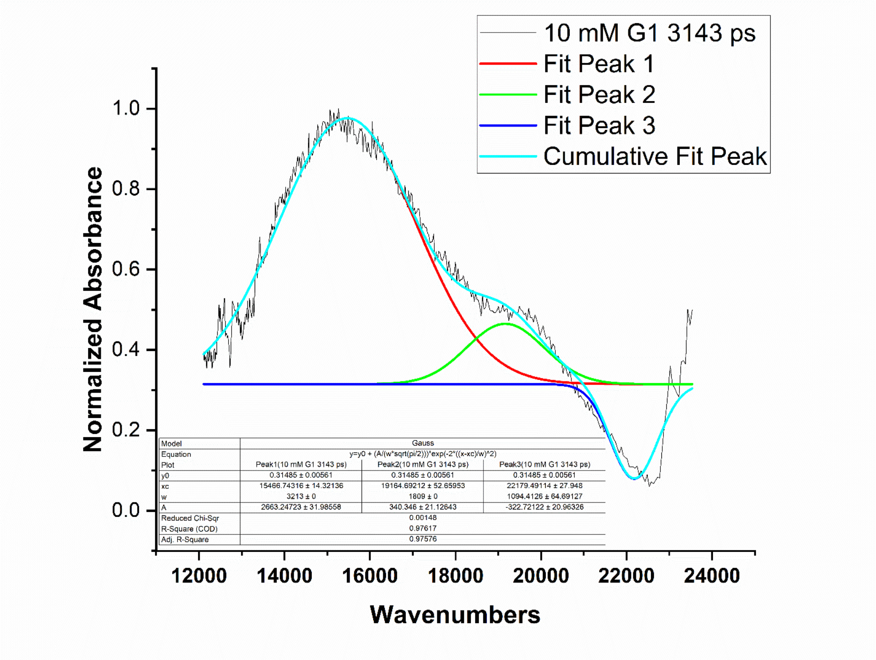
10 mM G1 3143 ps
Fit Peak 1
Fit Peak 2
Fit Peak 3
Cumulative Fit Peak
Normalized Absorbance
Wavenumbers
Model
Gauss
Equation
y=y0 + (A/(w*sqrt(pi/2)))*exp(-2*((x-xc)/w)^2)
Plot
Peak1(10 mM G1 3143 ps)
Peak2(10 mM G1 3143 ps)
Peak3(10 mM G1 3143 ps)
y0
0.31485 ± 0.00561
0.31485 ± 0.00561
0.31485 ± 0.00561
xc
15466.74316 ± 14.32136
19164.69212 ± 52.65953
22179.49114 ± 27.948
w
3213 ± 0
1809 ± 0
1094.4126 ± 64.69127
A
2663.24723 ± 31.98558
340.346 ± 21.12643
-322.72122 ± 20.96326
Reduced Chi-Sqr
0.00148
R-Square (COD)
0.97617
Adj. R-Square
0.97576

Following Figure: Triplet state absorption spectra of the 10, 50, and 400 mM samples after 485 nm excitation and Gaussian curve fits assuming two triplet state absorption bands and ground state depletion.

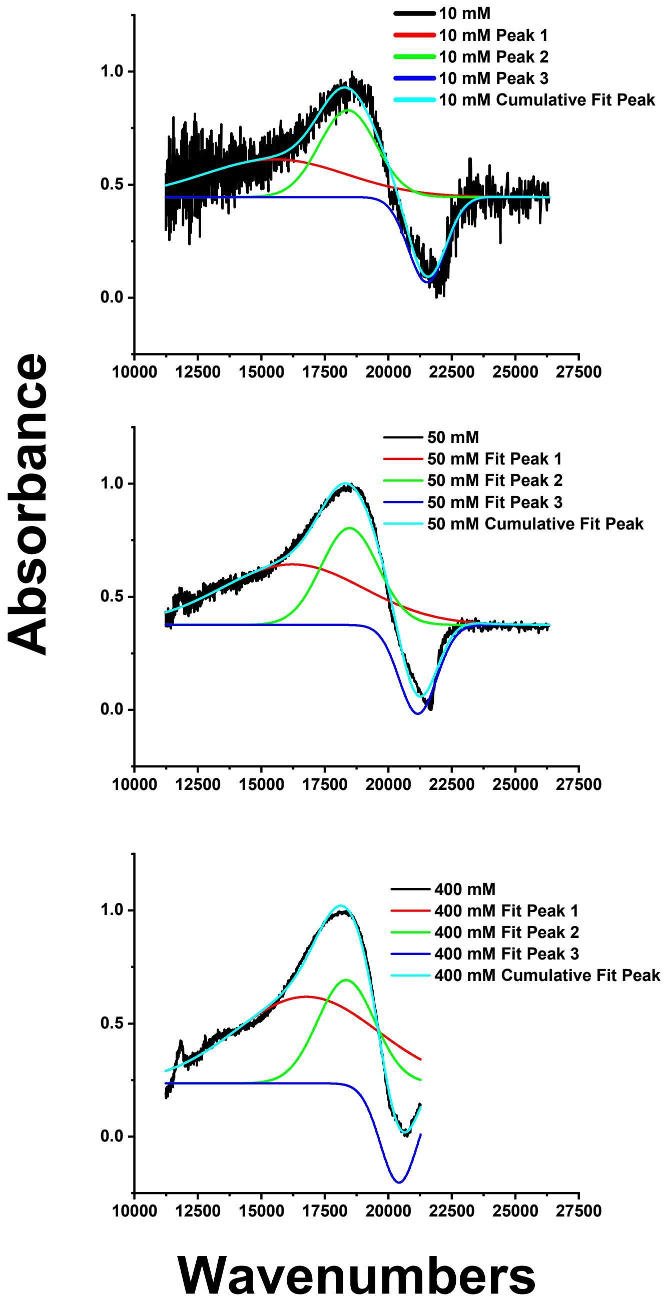

Supplement: Supplementary file 1 [file jp5c05728_si_001.pdf]
